# Circadian Oscillations in Branchlet Angle and Irradiance‐Dependent Acclimation in *Chara braunii* Under Continuous Light

**DOI:** 10.1002/pld3.70194

**Published:** 2026-10-05

**Authors:** Alena‐Maria Maidel, Katarina Kurtović, Carolin M. Heise, Stanislav Vosolsobě, Jan Petrášek, Stefan Timm, Hendrik Schubert

**Affiliations:** ^1^ Aquatic Ecology Department, Faculty of Mathematics and Natural Sciences University of Rostock Rostock Germany; ^2^ Department of Experimental Plant Biology, Faculty of Science Charles University Prague 2 Czech Republic; ^3^ Centre Algatech, Institute of Microbiology Czech Academy of Science Třeboň Czech Republic; ^4^ Plant Physiology Department, Faculty of Mathematics and Natural Sciences University of Rostock Rostock Germany

**Keywords:** endogenous regulation, metabolomics, phylogenetics, streptophytes

## Abstract

Endogenous biological rhythms are central to temporal organization in land plants, yet their evolutionary origins in streptophyte algae remain unresolved. We investigated whether rhythmic morpho‐physiological processes in *Chara braunii* are consistent with endogenous regulation and how continuous light intensity modulates these responses. In *C. braunii* , time‐lapse imaging, pigment quantification, chlorophyll fluorescence measurements, and metabolomic profiling were conducted under continuous low (dim) light and continuous higher (regular) light conditions. Circadian oscillations in branchlet angle persisted under continuous dim light but were attenuated under continuous regular light, whereas internodal elongation showed only limited temporal structure. Pigment and metabolite measurements, obtained once per day under two different continuous light conditions, revealed intensity‐dependent physiological adjustment, including changes in chlorophyll and carotenoid contents and altered accumulation of tricarboxylic acid cycle intermediates such as malate and fumarate. Chlorophyll fluorescence parameters showed no detectable temporal variation under the applied sampling scheme. Comparative genomic analyses identified putative homologs of several canonical land‐plant clock‐associated components, whereas canonical TOC1 and ZTL orthologs were not detected. Together, these results are consistent with the presence of endogenous regulatory components governing rhythmic outputs in *C. braunii* , while highlighting irradiance as a major modulator capable of suppressing these outputs. The parallel attenuation of rhythmic outputs and the shift toward a stress‐associated metabolic profile under higher irradiance suggest that higher irradiance affects both endogenous regulatory outputs and physiological acclimation responses, although whether these processes are causally linked remains to be determined.

## 1 Introduction

Rhythmic movements are widespread among photosynthetic organisms and can arise either from endogenous oscillators (circadian clocks) or from direct responses to recurring environmental cues such as light and temperature (Durand et al. 2024; Venkat and Muneer 2022). The concept that endogenous rhythms persist independently of external timing signals was first formally proposed by Bünning (1936). A defining property of circadian systems is their persistence under constant conditions (Lüning et al. 1997; Lüning 2001). Endogenously generated rhythms regulate diverse physiological processes in plants, including organ movements, growth, and stomatal behavior, and are thought to help coordinate cellular processes with predictable environmental fluctuations (Webb 2003).

The evolutionary origin of endogenous clock‐driven regulation is still unresolved. While complex transcriptional clock networks have been characterized in angiosperms (Alabadí et al. 2001; Cha et al. 2017; Más et al. 2003), it is still unclear when and how self‐sustained temporal coordination emerged during the transition from aquatic algal ancestors to land plants. However, the loss of observable rhythmicity under continuous light does not imply the absence of endogenous clock function but may result from irradiance‐dependent suppression of rhythmic outputs.

Charophytes occupy a pivotal phylogenetic position close to the base of land plants (Schubert, Bernhardt, et al. 2024) and therefore provide a unique evolutionary window into early streptophyte regulation. In *Characeae*, light intensity strongly influences branchlet angle and apical growth orientation (Schneider et al. 2006), yet the regulatory basis of these morphogenetic responses remains unresolved. Previous studies have primarily linked rhythmic morphogenetic outputs in charophytes (e.g., branchlet movements and growth‐related patterning) to signal transduction via plant hormones, particularly auxin (Beilby et al. 2015; Kurtović et al. 2025; Kwiatkowska et al. 1995; Lazar et al. 2013). However, it remained unclear whether these rhythms are attributable to an endogenous, self‐sustaining oscillator or represent direct responses to light intensity.

Beyond morphogenesis, continuous light imposes substantial photophysiological and metabolic demands. Irradiance level alters photosynthetic efficiency, which is balanced by adjusting light harvesting and photoprotection via changes in chlorophyll and carotenoid pools (Nunes‐Nesi et al. 2010; Proietti et al. 2021). Central metabolites of the tricarboxylic acid (TCA) cycle and the Calvin Benson Bassham (CBB) cycle participate in carbon metabolism and these fluxes are sensitive to changes in light intensity (Flis et al. 2016). These light intensity‐dependent acclimation processes are expected to operate independently of rhythmic control, yet may co‐occur with changes in rhythmic outputs under the same light conditions. It is therefore important to assess how organisms acclimate physiologically and metabolically to continuous light across different irradiances.

In land plants, the metabolic state interacts bidirectionally with endogenous rhythmic regulation—photosynthesis‐derived sugars, for instance, entrain core clock components (Dodd et al. 2015). This coupling links photosynthetic performance and carbon metabolism to rhythmic regulatory networks. In angiosperms, robust self‐sustained oscillations arise from interlocked transcriptional‐translational feedback loops comprising CCA1/LHY, TOC1/PRRs, ZTL‐GI‐mediated light input, and the evening complex (ELF3/ELF4/LUX) (Alabadí et al. 2001; Cha et al. 2017; Más et al. 2003; Nusinow et al. 2011). If comparable endogenous regulation operates in *Characeae*, it would imply the presence of functional clock‐associated components. Although homologs of several clock‐related genes have been identified in bryophytes (Holm et al. 2010; Linde et al. 2017) and lycophytes (Wickell et al. 2021), comparable evidence for streptophytes remains scarce, despite the availability of the fully sequenced genome of *C. braunii* C. C. Gmelin (Nishiyama et al. 2018). Consequently, the functional incorporation of clock‐associated components into coordinated rhythmic and metabolic regulation in early streptophyte lineages remains unresolved.

In this study, we investigated oscillations in branchlet angle in *C. braunii* under a regular photoperiod and continuous light conditions. To test for endogenous regulation of branchlet‐angle oscillations and to examine physiological acclimation to continuous light, we formulated two hypotheses: (i) branchlet‐angle oscillations in *C. braunii* are endogenously coordinated, persist under continuous light conditions, but are attenuated at higher irradiance; and (ii) continuous light at different intensities induces distinct acclimation responses in pigment and metabolite pools that co‐occur with differences in the persistence of branchlet‐angle oscillations.

## 2 Material and Methods

### 2.1 *C. braunii* Germination and Vegetative Propagation

A unialgal culture of *C. braunii* C. C. Gmelin, strain NIES‐1604, was obtained from the NIES collection (https://mcc.nies.go.jp/). A semiaxenic culture was established by germinating oospores as described in Kurtović et al. (2025). Four‐week‐old thalli containing mature oospores were harvested and air‐dried at room temperature for 1 month. The thalli were then rehydrated, and oospores were manually extracted using microforceps. To break the dormancy, oospores were placed in a refrigerator at 4°C for 4 months. Following stratification under sterile conditions, the oospores were washed with 1% Tween 20, sterilized with 10% commercial bleach for 8 min, and rinsed thoroughly with sterile water. The oospores were then sown on 0.5% plant agar (P1001; Duchefa Biochemie, Haarlem, the Netherlands). Germinated protonemata were transferred to a modified soil‐sand medium (SWCN‐4; https://mcc.nies.go.jp/medium/en/swcn4.pdf) in round‐bottom glass tubes. For the medium, 1 cm of garden soil was added to the bottom of each tube and moistened with a few drops of distilled water. This was overlaid with 4–5 cm of silica sand, double autoclaved and supplemented with sterile distilled water.

### 2.2 Cultivation Conditions

*C. braunii* cultures were precultivated for 2 weeks under aquarium light (F15W/T8 AQUASTAR, Cat No. 0002224; Sylvania) at room temperature. All subsequent experimental treatments (Figure 1), including light/dark and continuous light regimes, were conducted in a custom‐built imaging chamber equipped with LED strips (construction details in Supporting Information; Kurtović et al. 2025; Kurtović et al. 2026).

**FIGURE 1:**
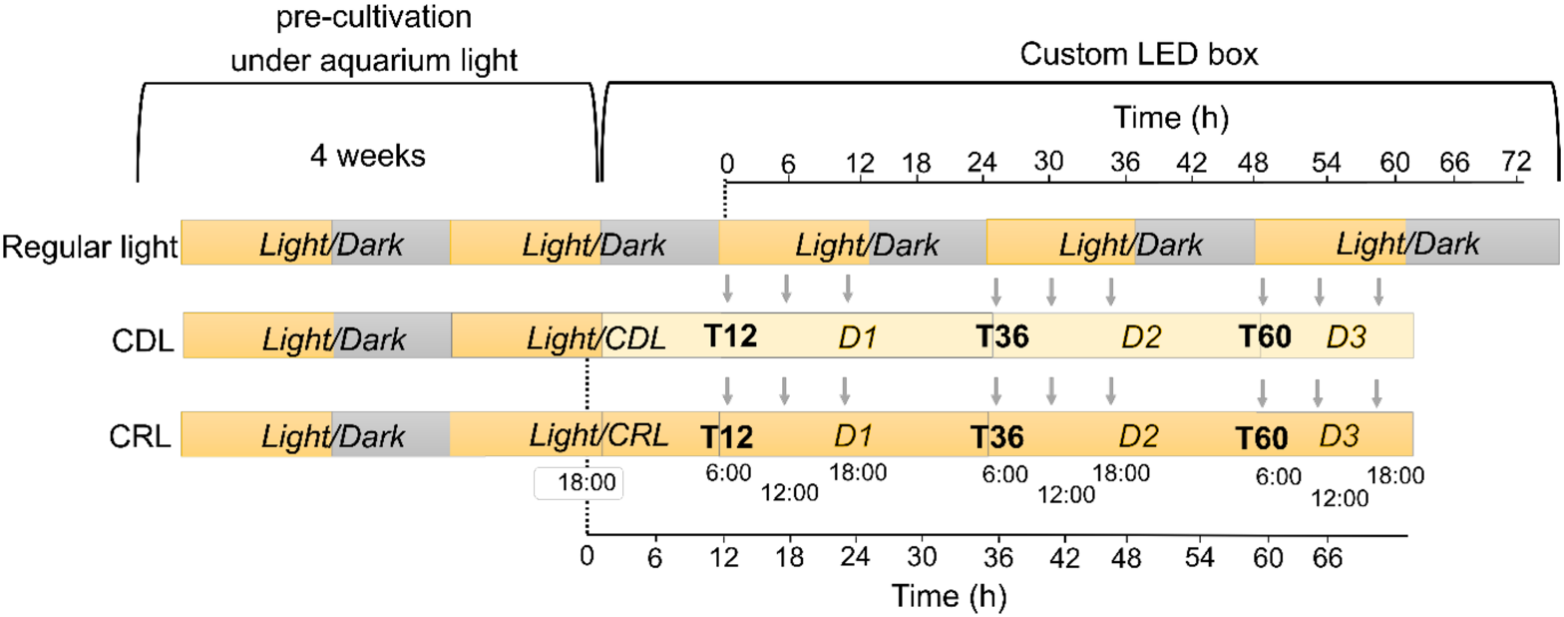
Schematic overview of the experimental design. *Chara braunii* cultures were precultivated under aquarium light in a 14‐h light (30 μmol photons m^−2^ s^−1^) /10‐h dark photoperiod before transfer to a custom‐built LED box. For continuous light experiments, cultures were transferred at 18:00 to either continuous dim light (CDL, 15 μmol photons m^−2^ s^−1^) or continuous regular light (CRL, 30 μmol photons m^−2^ s^−1^). After 12 h (T12), 36 h (T36), and 60 h (T60), pigment and metabolite samples were collected at 6:00. Day 1 (D1), Day 2 (D2), and Day 3 (D3) refer to sampling days following transfer to continuous light conditions. Gray arrows indicate time points at which photosynthesis measurements were performed (6:00, 12:00, and 18:00 on D1–D3).

For branchlet dynamics under entrained conditions, *C. braunii* cultures were maintained under a 14‐h light/10‐h dark cycle (lights on at 5:00, lights off at 19:00). Branchlet angles were quantified manually during the light phase at 6:00, 12:00, and 18:00, with 6:00 defined as the first measurement time point for the light/dark dataset (Figure 1, Figure 2). Due to technical limitations, time‐lapse imaging during the dark phase was not feasible, as continuous monitoring would have required infrared or specialized low‐light imaging systems.

**FIGURE 2:**
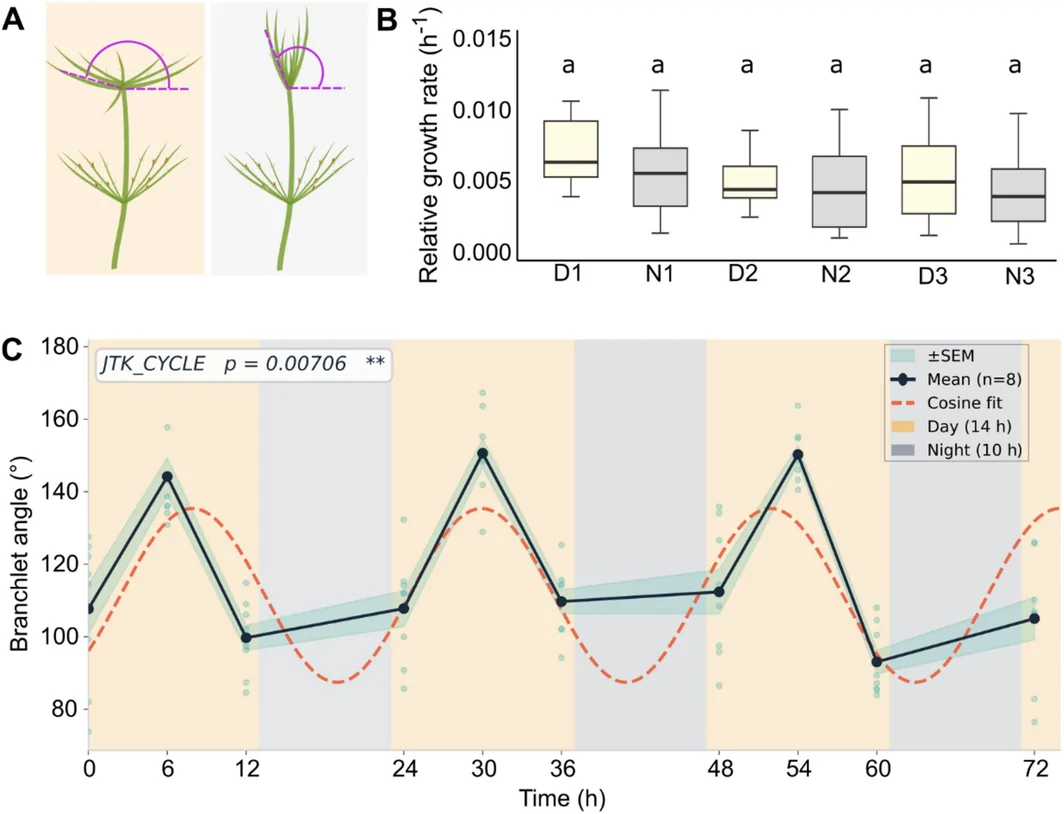
Rhythmicity of branchlet angles in *Chara braunii* recorded over 72 h under a 14‐h light/10‐h dark photoperiod. (A) Schematic representation of *C. braunii* thallus containing the first two nodes. The left drawing (shaded in yellow) shows a first node with open branchlets during the day, while the right drawing (shaded in gray) shows a node with closed branchlets during the night. (B) Boxplot of relative growth rates measured during the day and night phases across three consecutive light/dark cycles. No significant differences were detected among the groups (*p* > 0.05, letters indicate homogeneous groups based on statistical test). Growth occurred consistently across day and night periods. (C) Branchlet angle dynamics of *C. braunii* over a 72‐h period under 14‐h light/10‐h dark photoperiod. Branchlet angles of *Chara* were measured at 6:00, 12:00, and 18:00, over a 12‐h light (30 μmol photons m^−2^ s^−1^) phase (mean ± SD; *n* = 8 independent biological replicates). Shaded areas indicate day (yellow) and night (gray) phases, with time 0 corresponding to 6:00, one hour after light onset at 5:00; the light phase ends at 19:00. Individual replicates are represented in green dots, and the population mean ± SEM in light green shading. The dashed magenta line represents a fitted 24‐h cosine model visually describing the rhythmic oscillation of branchlet angle. JTK_CYCLE analysis detected significant rhythmicity (*p* = 0.007, **p < 0.01), with a consistent midday peak indicating maximum branchlet opening around noon. Replicate‐level JTK_CYCLE results and FDR‐adjusted *p* values are provided in Table S4.

For continuous light treatments, cultures were transferred from the entrained regime to continuous illumination at 18:00 (time 0; Figure 1). Time‐lapse imaging was conducted in the custom box with either continuous dim light (CDL, 15 μmol photons m^−2^ s^−1^, Figures 1 and 3D) or continuous regular light (CRL, 30 μmol photons m^−2^ s^−1^, Figures 1 and 3B) and a constant temperature of 21.5°C. The spectral composition of both the aquarium and LED lighting was measured using a spectroradiometer SR9910‐PC (Macam Photometrics Ltd., Livingstone, Scotland) equipped with a 4π spherical sensor and is shown in Figure S1.

**FIGURE 3:**
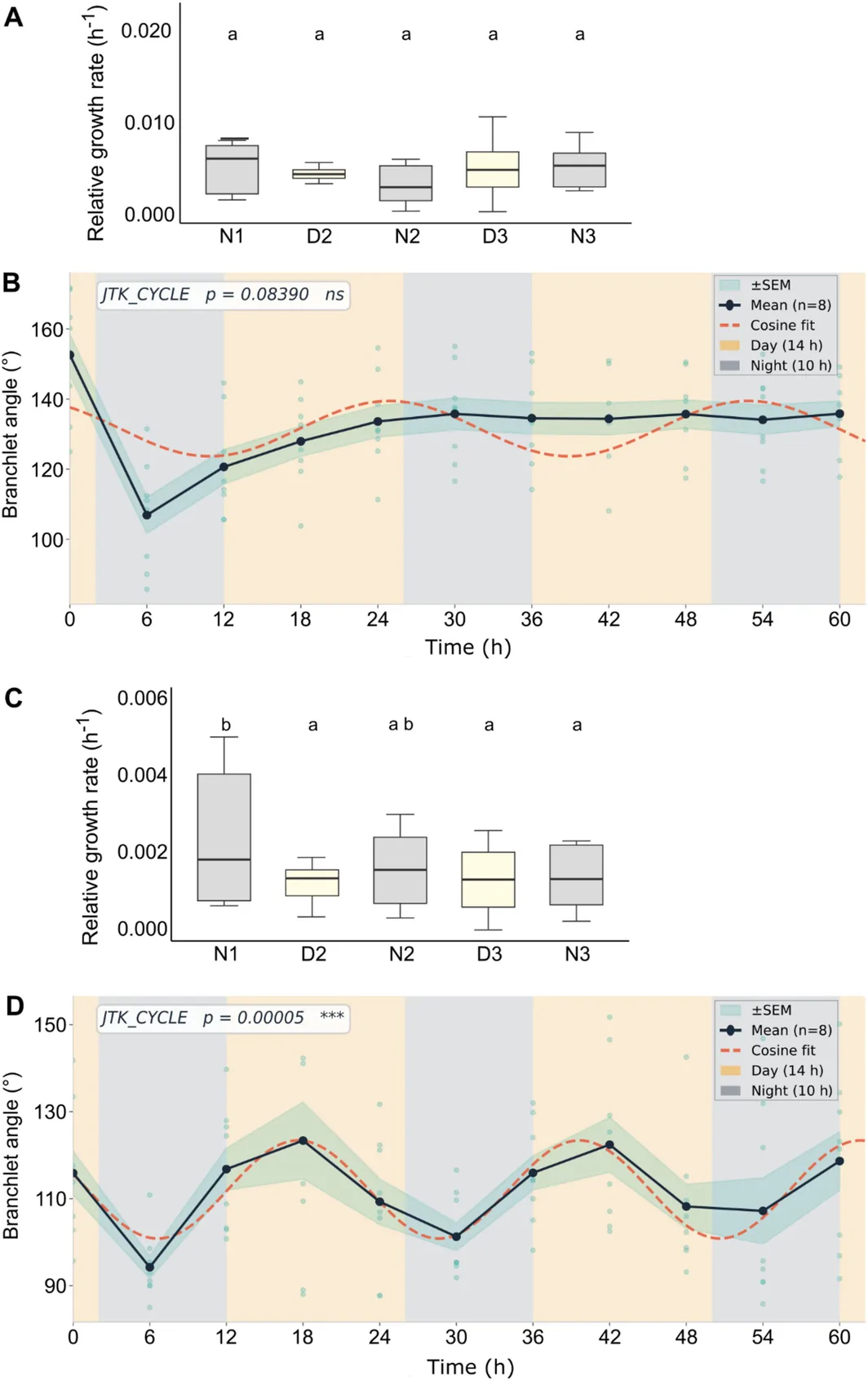
Branchlet angle dynamics and growth rate in *Chara braunii* under continuous light conditions. (A) Boxplot of relative growth rates of the first internodal cell under continuous regular light (CRL, 30 μmol photons m^−2^ s^−1^) conditions. No significant differences were detected among the groups (*p* > 0.05, letters indicate homogeneous groups based on a linear mixed‐effects model followed by Tukey‐adjusted post hoc comparisons). (B) Branchlet angle dynamics of *C. braunii* over a 60‐h period under continuous regular light. Branchlet angles of *Chara* were measured at 6‐h intervals (mean ± SEM; *n* = 8 independent biological replicates). Thalli initially showed a closing movement followed by a transition to a semiopen, stable position over time. (C) Boxplot of relative growth rates of the first internodal cell under continuous dim light (CDL, 15 μmol photons m^−2^ s^−1^) conditions. Different letters indicate statistically significant differences. (D) Branchlet angle dynamics of *C. braunii* over a 60‐h period under continuous dim light. Branchlet angles of *Chara* were measured at 6‐h intervals (mean ± SEM; *n* = 8 independent replicates). Under continuous dim light, branchlets show rhythmic opening and closing. (B, D) Shaded areas indicate projected subjective light (yellow) and dark (gray) phases based on the preceding entrainment regime. Time 0 corresponds to the end of the light phase (18:00) prior to transfer to continuous light conditions. Individual replicates are represented by light green dots, and the population mean ± SEM in light green shading. The dashed red line represents a best fitted 24‐h cosine curve describing the rhythmic oscillation of branchlet angle. JTK_CYCLE analysis did not detect statistically significant rhythmicity (*p* = 0.084, ns) for CRL conditions (B), while CDL conditions (D) resulted in significant rhythmicity (*p* = 0.00005, ***p < 0.001). Replicate‐level JTK_CYCLE results and FDR‐adjusted *p* values are provided in Table S4.

### 2.3 Timelapse Imaging

Time‐lapse imaging of *C. braunii* under continuous light was performed using a custom setup. Two‐week‐old *Chara* cultures in glass test tubes were placed in a 3D‐printed stand inside a small open‐top cardboard box (25 × 20 × 15 cm) lined with black fabric to reduce reflection from glass. The box was positioned within a cultivation chamber box to either (1) a 14‐h light/10‐h dark cycle, (2) regular, or (3) dim light intensity, and a smartphone aligned with a small hole in the box to capture images at 10‐min intervals over a 3‐day period. The camera was stabilized with silver tape, and a white background was used behind the samples to further reduce reflections. The standard photoperiod experiment was started in the morning (6:00) because the imaging setup requires the acquisition to begin during the daytime (nighttime imaging is not possible with this system). Continuous light conditions were initiated after the completion of the preceding light period in the regular photoperiod (18:00).

### 2.4 Measurement of Opening Angle and Relative Growth

Time‐lapse images were imported into Fiji (Version 2.9.0) as image sequences. For photoperiod conditions (14‐h light/10‐h dark cycle), three timepoints were selected for analysis (6:00, 12:00, and 18:00), while under continuous light, four timepoints (6:00, 12:00, 18:00, and 24:00) were used. Eight *C. braunii* thalli were tracked in each condition. The angles between the branchlet tip and the basal node were measured consistently on the same side of each thallus using the angle measuring tool in ImageJ, with 90° defined as the closed (upright) position. Cell length was measured from time‐lapse images in Fiji, and relative growth rate (RGR) was calculated between consecutive time points using RGR = (ln L_2_ − ln *L*_1_) / (*t*_2_ − *t*_1_) and expressed in h^−1^.

### 2.5 Photosynthesis Parameters

Photosynthesis parameters were measured at 6:00, 12:00, and 20:00 on the first, second, and third day following transfer to continuous light (D1–D3, Figures S3 and S4). Measurements were performed utilizing a pulsed‐amplitude modulation (PAM) chlorophyll fluorometer (DUAL‐PAM‐100; Heinz Walz GmbH, Effeltrich, Germany). Prior to the measurement process, the organisms were subjected to a 30‐min dark adaptation period. Subsequently, photosynthesis‐irradiance (PI) curves were generated using the following procedure. The organisms were transferred to a quartz cuvette with the specific culture medium. PI curves were constructed encompassing 17 successive actinic light irradiation steps (6, 15, 28, 52, 78, 107, 149, 203, 272, 357, 455, 569, 722, 894, 1110, 1723, and 3242 μmol photons m^−2^ s^−1^). The duration of each irradiance was set at 30 s for the initial four steps and 50 s for the subsequent steps. The calculation of photosynthetic parameters was conducted utilizing the Dual PAM software (Version 3.21, Heinz Walz GmbH). The dark‐adapted PSII yield (Y_dark_ (II)) was recorded and the initial slope (α) and the light saturation point (I_k_) were calculated by fitting the PI curves to the model of Eilers and Peeters (1988) with the Excel (Microsoft Office, LTSC Professional Plus 2021) solver add‐in with the least squares method.

### 2.6 Pigment Analysis

Transfer to continuous light occurred at 18:00 (time 0; Figure 1), when the onset of the dark phase would normally have occurred. Until then, plants had been maintained under normal daytime light conditions. Pigment and metabolite samples were collected at 6:00 after 12 h (T12), 36 h (T36), and 60 h (T60) of continuous light (Figure 1). For pigment analysis, samples were frozen and analyzed simultaneously. Prior to measurement, the samples were thawed and 6 mL of N,N‐dimethylformamide (DMF; Carl Roth GmbH, Karlsruhe, Germany) were added. The samples were then subjected to an incubation process in conditions of darkness and at a temperature of 4°C for a period of approximately 24 h. Without transferring the specimen, the remaining liquid was transferred into a 5 mL quartz cuvette. An absorption wavelength scan was then conducted using the spectrophotometer DR3900 (Version 1.1, Hach Lange, Düsseldorf, Germany). The concentration of the extracted pigments, namely chlorophyll *a* (Chl *a*), chlorophyll *b* (Chl *b*), and carotenoids (Car), was calculated according to the equation given by Wellburn (1994).

### 2.7 Metabolite Quantification

For LC–MS/MS analysis, 50–100 mg *Chara* thalli were harvested, shock frozen in liquid nitrogen, and stored at −80°C until further analysis. The extraction was essentially performed as described by Reinholdt et al. (2019) with slight modifications. The samples were shock frozen a second time before the final lyophilization step. The dried extracts were then dissolved in 400 μL LC–MS grade water (Roth, Germany), shaken for 30 min and filtered through 0.45 μm filters (Chromafil AO‐45/3, MACHEREY‐NAGEL GmbH & Co, KG; Düren; Germany). Four μL of the filtrate were analyzed on the ultra‐high performance liquid chromatograph (UHPLC) tandem mass spectrometer LC–MS/MS‐8050 system (Shimadzu) and the incorporated method package for primary metabolites (Version 2, Shimadzu). Analysis and identification of 35 compounds related to primary metabolism were performed as described previously (Reinholdt et al. 2019).

### 2.8 Phylogenetic Analysis

Sequences of canonical circadian‐clock proteins in *A. thaliana* (Table S3) were obtained on the Arabidopsis Information Resource (TAIR, Rhee et al. 2003) and used for National Center for Biotechnology Information (NCBI) Basic Local Alignment Search Tool (BLAST) search (Camacho et al. 2023) in *C. braunii* proteome (GCA_003427395.1) and in Phycocosm database (Grigoriev et al. 2021) or OneKP database (Leebens‐Mack et al. 2019). Putative homologs were verified using a reverse BLAST search on TAIR. Transcriptomic datasets were used only as evidence of gene presence. The absence of a transcript was not interpreted as evidence of gene loss.

### 2.9 Data Analysis

All statistical analyses were performed in R Version 4.3.1 (R Core Team 2023), using the packages *car*, *lattice* (Sarkar 2008), and *vegan* (Oksanen et al. 2013). The significance threshold was defined as α = 0.05. Due to limited biological replication (*n* = 2), photosynthesis data were interpreted descriptively. For pigment data, comparisons were conducted between light treatments (CRL vs. CDL) for each respective time point (T12, T36, and T60). For metabolite data, comparisons were made between time points (T12 vs. T36 and T12 vs. T60) within each light treatment group (CRL and CDL).

Rhythmicity of branchlet‐angle time series was assessed using the JTK_CYCLE algorithm (Hughes et al. 2010), a nonparametric statistical method based on the Jonckheere–Terpstra–Kendall test. For each dataset, the analysis was performed on the mean time series (averaged across all eight replicates at each time point). JTK_CYCLE was also applied to each replicate series individually, and resulting *p* values were corrected for multiple testing using the Benjamini–Hochberg false discovery rate (FDR) procedure via p.adjust (method = “BH”). A rhythm was considered statistically significant at BH‐adjusted *p* < 0.05. To visualize the fitted rhythm, a cosine model of the form A·cos (2π(*t* − *φ*) / *T*) + *C* was fitted to the mean time series using robust nonlinear least squares optimization (nlsLM from the minpack.lm R package; Elzhov et al. 2023). Replicate‐level JTK_CYCLE analyses, including FDR‐adjusted *p* values, are provided in Table S4.

The differences in relative growth of *Chara* were calculated over 12‐h time intervals. A linear mixed‐effects model was fitted using the lmer() function from the lme4 package (Bates et al. 2015), with interval as a fixed effect and replicate as a random effect to account for repeated measurements. The significance of the fixed effect was assessed using the Satterthwaite approximation for degrees of freedom provided by the lmerTest package. Post hoc pairwise comparisons between intervals were conducted using the emmeans package (Lenth 2023) with Tukey's honest significant difference correction for multiple testing. Statistically similar groups were assigned letters using compact letter display (CLD) via the multcomp package (Hothorn et al. 2008) and visualized on boxplots generated with ggplot2.

Pigment and metabolite analyses were each performed using six independent biological replicates per light treatment and sampling time. For statistical analysis, pigment and metabolite concentrations were log₁₀‐transformed. Normality was assessed using the Shapiro–Wilk test, and homogeneity of variances using Levene's test. For pigment comparisons, CDL and CRL were compared separately at T12, T36, and T60 using Welch's two‐sample *t*‐tests. The corresponding test statistics, degrees of freedom, and *p* values are reported in Table S5. For each metabolite comparison, either a Student's *t*‐test, Welch's *t*‐test, or Wilcoxon rank‐sum test was selected according to the results of the normality and variance‐homogeneity assessments. The specific test and corresponding *p* value for each metabolite comparison are reported in Table S6. Additionally, metabolite concentrations were normalized within each metabolite and light treatment group to the mean value of T12, enabling relative comparisons across time points. Metabolites were grouped into amino acid and organic acid subgroups (Table S1) and normalized to their respective T12 values (Table S2). Statistical differences between amino acid and organic acid groups within each light treatment and sampling time were assessed using Wilcoxon rank‐sum tests with BH adjustment. Furthermore, fold changes (FCs) were calculated as the ratio of mean intensities at T36 or T60 relative to T12 and are presented on a log_2_ scale. Log_2_ transformation was applied exclusively for visualization purposes, not for statistical testing. To facilitate interpretation of temporal metabolic changes, mean normalized concentrations were log_2_‐transformed, and single‐metabolite heatmaps were generated based on these values. A metabolic map was constructed following Schweiger et al. (2014) using the Kyoto Encyclopedia of Genes and Genomes (KEGG) reference pathways (Kanehisa and Goto 2000). Heatmaps were generated using the R package pheatmap (Kolde 2019).

Because branchlet‐angle oscillations, photosynthetic parameters, pigments, and metabolites were assessed using different sampling schedules and temporal resolutions, direct temporal integration across these datasets was limited. Cross‐assay similarities were therefore interpreted as treatment‐associated co‐occurrence rather than as evidence of synchronous or causally linked responses.

Tables S2–S6 can be found in the separate Excel file (Supplemental_Tables.xlsx).

Video S1, S2, and S3 can be found in the separate video files (Day_X_scalebar.avi); *X* = 1, 2 or 3.

## 3 Results

### 3.1 Rhythmic Oscillations in Branchlet Angle Under Entrained Light/Dark Conditions

Light intensity was shown to affect branchlet movement in *Chara intermedia* and *Chara hispida* , where under high light conditions, branchlets grew steeply upwards, while under lower light, they adopted a horizontal orientation (Schneider et al. 2006). However, in young *C. braunii* thalli, we observed that the upper branchlets exhibited rhythmic movement (Figure 2A). To confirm this observed phenomenon, we conducted time‐lapse imaging over a 72‐h period using young, actively growing *C. braunii* thalli (Video S1). To determine whether the regular photoperiod influences growth dynamics in *C. braunii* , we first quantified the elongation rate of the first internodal cell during both day and night phases. Measurements taken over three consecutive light/dark cycles (72‐h period) revealed no significant difference in relative growth rate between light and dark periods (Figure 2B). Thus, under the specific photoperiod and experimental conditions applied here, no effect on internodal elongation rate was detected.

In addition to the characteristic spiral growth previously described in *Nitella* (Green 1954), we observed a reproducible nutational movement of the apical branchlets over a 24‐h cycle (Figure 2C). Specifically, branchlets exhibited a coordinated opening and closing motion at the top node of the growing axis. Branchlet angles were measured at the following three time points: 6:00, 12:00, and 18:00, over a 12‐h light phase (Videos S1–S3), with the initial start timepoint corresponding to 6:00.

In the morning, following light onset, branchlets remained in a nearly vertical position before initiating an opening movement, reaching angles between 130° and 180°. By the evening, approximately 1 h before the dark period, the branchlets returned to a more vertical orientation with angles between 90° and 110°. Although measurements under the light/dark cycle were restricted to the light phase, JTK_CYCLE detected significant rhythmicity in the mean branchlet‐angle time series (*p* = 0.007). All eight replicate series remained significant after BH correction (Table S4), demonstrating robust entrained rhythmicity under the applied light/dark cycle. This rhythmic pattern contrasts with previous reports in other *Chara* species, where branchlet closure has been interpreted primarily as a protective response to high light stress (Schneider et al. 2015).

### 3.2 Irradiance‐Dependent Modulation of Branchlet‐Angle Oscillations Under Continuous Illumination

To test whether branchlet movement persists in the absence of an external dark phase, *C. braunii* thalli maintained under a 14‐h light/10‐h dark photoperiod were transferred to continuous light conditions. Under CRL (30 μmol photons m^−2^ s^−1^) and CDL (15 μmol photons m^−2^ s^−1^), branchlet angles were monitored using the same time‐lapse setup described above (Figure S2). In the absence of a dark phase, branchlet dynamics were observed continuously across the experimental period.

Internodal elongation continued under CRL; however, no significant differences were detected between projected subjective day and night intervals (Figure 3A). Angle measurements were obtained at 6‐h intervals (18:00, 0:00, 6:00, and 12:00) with time zero corresponding to 18:00 (Figure 1). Following transfer to CRL, thallus tips exhibited an initial closing movement during the first 24 h. Subsequently, branchlets partially reopened but stabilized in a semiopen state for the duration of the experiment (Figure 3B). JTK_CYCLE analysis of branchlet movement did not detect statistically significant circadian oscillation (*p* = 0.084); moreover, none of the replicate series remained significant after FDR correction (Table S4).

To assess whether lower irradiances influence the persistence of movement dynamics, a second set of *C. braunii* thalli was placed under CDL conditions (15 μmol photons m^−2^ s^−1^), commonly used in circadian rhythm studies in plants (Velez‐Ramirez et al. 2011). Under CDL, internodal elongation differed between projected subjective day and night intervals (Figure 3C), with the highest elongation rate observed during the first projected subjective night following transfer. Although the absolute opening angles were reduced relative to those observed under entrained light/dark conditions, the branchlets under CDL still displayed sustained and significant (*p* = 0.00005) periodic opening and closing dynamics across the 60‐h recording period (Figure 3D). However, only six out of eight replicate series remained significant after FDR correction (Table S4).

Together, these observations indicate that branchlet‐angle oscillation scan persist under CDL but are strongly attenuated under higher continuous irradiance. In the context of our experimental design, we aimed to test whether the observed oscillations are regulated by an endogenous mechanism in *C. braunii* . The data are consistent with an endogenous regulatory component whose observable output is modulated by light intensity.

### 3.3 Continuous Light Exposure Alters Pigment Accumulation in *C. braunii*

Continuous light (approximately 110 μmol photons m^−2^ s^−1^ PAR) has been shown to affect pigment distribution in vascular plants with studies reporting local pigment loss and compensation mechanisms (Globig et al. 1997). To assess the effect of continuous light exposure on pigment accumulation in *C. braunii*, thalli were transferred to either CDL (15 μmol photons m^−2^ s^−1^) or CRL (30 μmol photons m^−2^ s^−1^). Pigment concentrations were measured at three time points after transfer to continuous light, corresponding to 12 h (T12), 36 h (T36), and 60 h (T60; Figure 1). Because samples were collected only once every 24 h, at 6:00, these measurements reflect acclimation dynamics rather than potential circadian oscillations.

At T12 only, the chlorophyll *a* (Chl *a*) /chlorophyll b (Chl *b*) ratio was significantly higher in CDL‐treated thalli, whereas absolute concentrations of Chl *a*, Chl *b*, and carotenoids (Car) did not differ between treatments (Figure 4A–D). After 36 h of transfer (T36), pigment accumulation increased markedly under CDL, with significantly elevated concentrations of Chl *a*, Chl *b*, and Car compared to CRL. At T60, pigment levels declined slightly, and only the Chl *a*/Chl *b* ratio remained significantly higher under CDL, while Chl *a*, Chl *b*, and Car concentrations no longer differed between treatments. These results indicate treatment‐specific differences in pigment accumulation, with the strongest differences in absolute pigment concentrations observed at T36.

**FIGURE 4:**
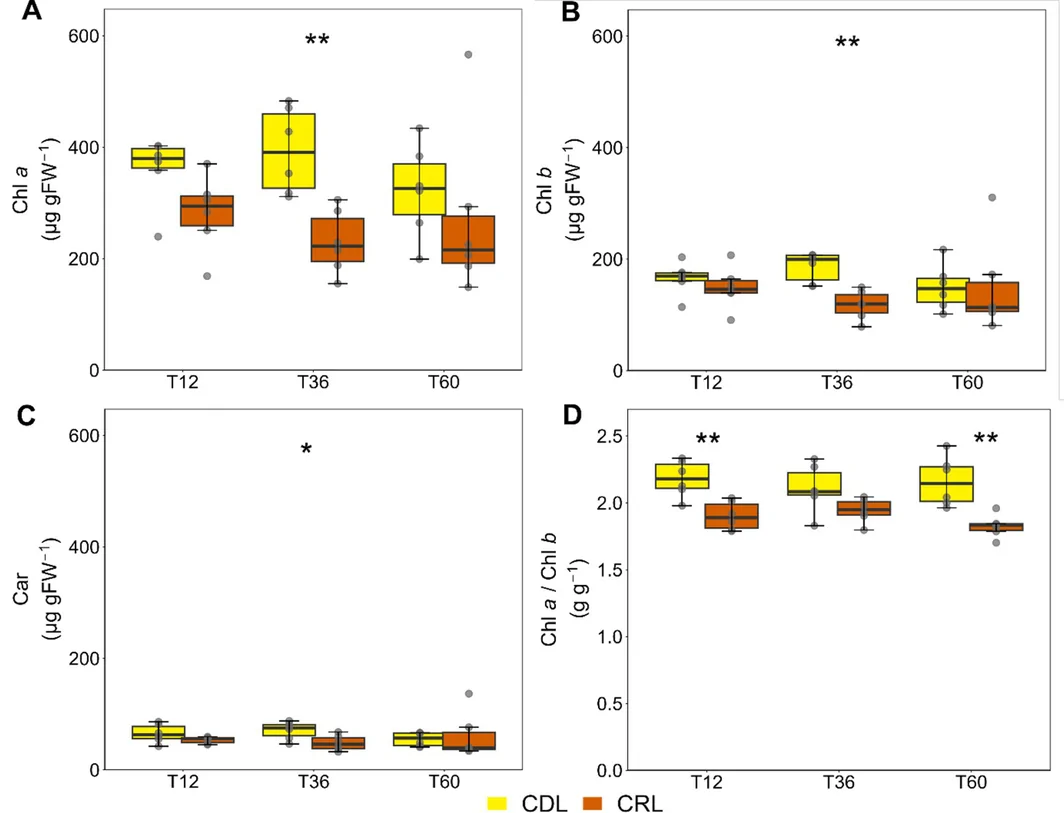
Pigment concentrations and ratios in *Chara braunii* under continuous light conditions. *C. braunii* thalli were transferred to continuous dim light (CDL, 15 μmol photons m^−2^ s^−1^) and continuous regular light (CRL, 30 μmol photons m^−2^ s^−1^) and sampled at 6:00 on three consecutive days following transfer, corresponding to 12 h (T12), 36 h (T36), and 60 h (T60). Panels show (A) Chlorophyll *a* (Chl *a*), (B) Chlorophyll *b* (Chl *b*), (C) Carotenoids (Car), and (D) Chl *a*/Chl *b* ratio. Data are given as boxplots with interquartile ranges (IQR; boxes), medians (horizontal lines), whiskers extending to the most extreme data points within 1.5 × IQR, and individual raw values (gray dots; *n* = 6 independent biological replicates). Statistical analyses were performed on log₁₀‐transformed data. Asterisks indicate statistically significant differences between light treatments within each sampling time, assessed using Welch's two‐sample *t*‐tests (*p* < 0.05 and **p < 0.01); the absence of asterisks indicates no statistically significant difference. The corresponding test statistics, degrees of freedom, and *p* values are provided in Table S5.

Pigmentation in *Chara* species is known to respond to irradiance, particularly through adjustments in Chl *b* and carotenoid levels (Maidel and Schubert 2026). Our data demonstrate that under continuous illumination, pigment pools respond dynamically to light intensity and exposure duration. Photosynthetic measurements, by contrast, did not reveal detectable temporal variation over the course of the day or across consecutive days within the temporal resolution of our sampling (Figures S3 and S4). Because pigment samples were collected only once per day at 06:00, these data primarily reflect changes across sampling days and are not directly comparable to the photosynthetic measurements with respect to within‐day variation.

### 3.4 Metabolite Accumulation Under Continuous Light Differs Between Light Intensities

Metabolite profiles can be influenced by light intensity (Bulut et al. 2025; Jänkänpää et al. 2012) and prolonged illumination (Proietti et al. 2021; Velez‐Ramirez et al. 2011). To investigate whether such responses occur in *C. braunii* under the present experimental conditions, thalli cultivated under a light/dark photoperiod were analyzed following transfer to continuous light (CDL, CRL). Metabolite concentrations were normalized to the respective T12 values (12 h after transfer). In total, 35 metabolites associated with primary metabolism were consistently detected across all samples (Table S1). Under CDL, several metabolites related to carbon and nitrogen metabolism showed increased normalized levels at T36 and T60 compared to T12, whereas, under CRL, changes were generally smaller in magnitude and more variable (Figure 5A). For visualization, metabolites were also grouped into compound classes (amino acids and organic acids; Figure 5B). Under CDL, no significant differences were detected between compound classes across time points. Under CRL, amino acids exhibited significantly higher normalized levels than organic acids at T36 and T60. These data indicate that continuous illumination induces treatment‐specific shifts in primary metabolite pools, consistent with irradiance‐dependent metabolic adjustment over time.

**FIGURE 5:**
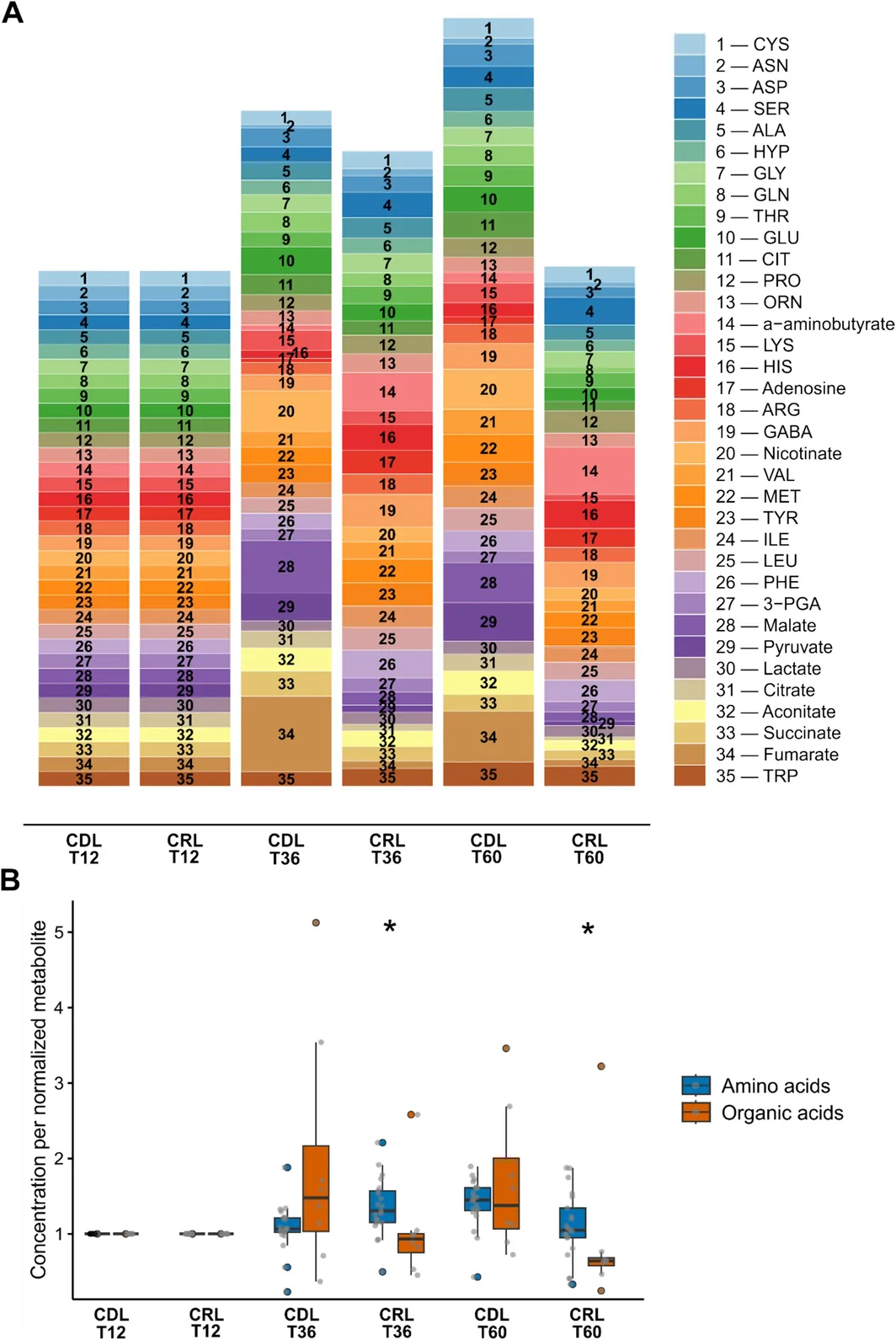
Metabolite concentrations in *Chara braunii* under continuous light conditions. *C. braunii* thalli were transferred to continuous dim light (CDL, 15 μmol photons m^−2^ s^−1^) and continuous regular light (CRL, 30 μmol photons m^−2^ s^−1^) and sampled at 6:00 on three consecutive days following transfer, corresponding to 12 h (T12), 36 h (T36), and 60 h (T60). Metabolite concentrations were normalized to the respective T12 values within each light treatment. T12 therefore represents the normalization baseline for both CDL and CRL. (A) Mean normalized concentration of individual metabolites (*n* = 6 individual biological replicates). (B) Mean normalized concentrations grouped by compound class (amino acids, organic acids; Table S1). Data are shown as box‐whisker plots, with boxes representing interquartile ranges (IQR), horizontal lines indicating medians, whiskers extending to the most extreme data points within 1.5 × IQR, and gray dots showing individual normalized metabolite values. Asterisks indicate significant differences between amino acid and organic acid groups within each light treatment and sampling time (Wilcoxon test; *p < 0.05, **p < 0.01, ***p < 0.001) while the absence of asterisks indicates no statistically significant difference. Metabolite abbreviations correspond to the legend; full names and classifications are provided in Tables S1 and S2.

### 3.5 Specific Metabolites Show Treatment‐Dependent Changes Under Continuous Dim and Regular Light

Specific metabolite pools showed treatment‐ and time‐dependent changes under continuous illumination. Figure 6 presents log_2_ FCs of selected metabolites relative to T12 (12 h after transfer) under CDL and CRL. Under CDL, malate, fumarate, nicotinate, and glutamate (GLU) showed significantly higher normalized levels at T36 compared to T12. In contrast, adenosine and histidine (HIS) were significantly lower at T36. At 60 h after transfer (T60), malate, fumarate, and nicotinate remained significantly elevated relative to the values after 12 h of transfer (T12), and alanine additionally showed a significant increase.

**FIGURE 6:**
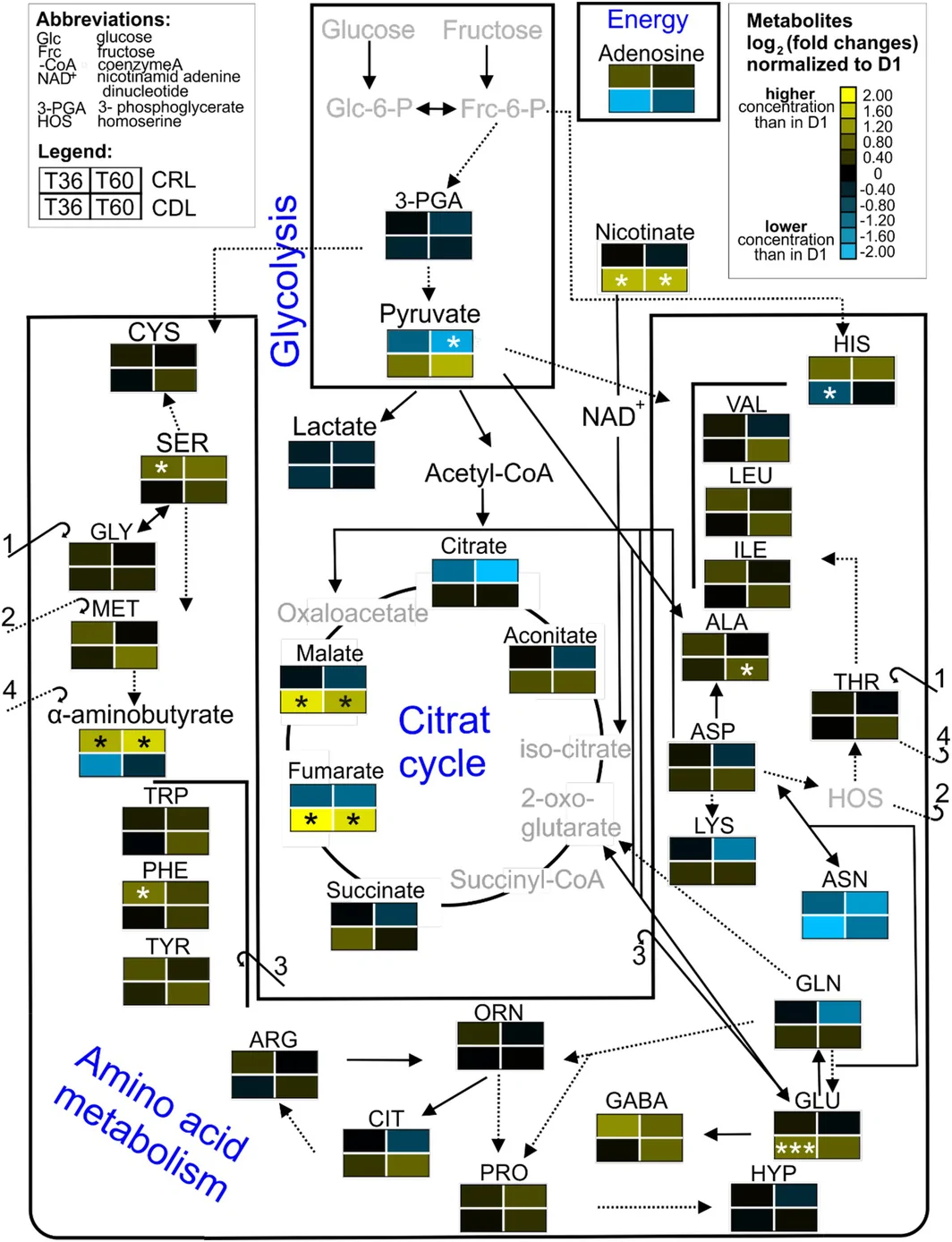
Metabolic pathway map showing log_2_ fold changes in selected metabolite levels in *Chara braunii* under continuous illumination. *C. braunii* thalli were transferred to continuous dim light (CDL, 15 μmol photons m^−2^ s^−1^) and sampled at 6:00 on three consecutive days following transfer, corresponding to 12 h (T12), 36 h (T36), and 60 h (T60). T12 represents the normalization baseline within each light treatment. Yellow indicates increased metabolite levels relative to T12, while blue indicates decreased levels (mean of 6 independent biological replicates). Not all reactions and intermediates are shown; dashed arrows denote omitted steps. Curved arrows indicate the same process as straight arrows but are redirected for clarity and are marked with identical numbering. Metabolites shown in gray were not analyzed. Asterisks indicate significant differences compared to T12 within each condition (Wilcoxon tests, Welch's *t*‐tests or Student's *t*‐tests; *p < 0.05, **p < 0.01, ***p < 0.001). Absence of asterisks indicates no statistically significant difference relative to T12. Full metabolite names are provided in Table S1. The statistical test used for each comparison and the corresponding *p* value are provided in Table S6.

Under CRL conditions, serine (SER), phenylalanine (PHE), and α‐aminobutyrate were significantly increased 36 h after transfer relative to 12 h after transfer, while α‐aminobutyrate remained elevated at 60 h after transfer. In contrast, pyruvate levels were significantly reduced at T60 (Figure 6). No significant changes were detected for other metabolites, including tryptophan (TRP) across time points. These patterns indicate treatment‐specific shifts in individual metabolites under continuous illumination, consistent with irradiance‐dependent metabolic adjustment.

### 3.6 Homologs of Land‐Plant Clock‐Associated Genes Are Present in *C. braunii*

Next, we analyzed the presence of clock‐associated genes in *C. braunii* and all annotated Streptophyta algae genomes deposited in the Phycocosm database (Grigoriev et al. 2021). *C. braunii* contains homologs of several major land‐plant clock‐associated gene families (Figure 7), though with a remarkably different degree of conservation compared to flowering plants. The REVEILLE (RVE) family is represented by only one homolog with the characteristic N‐terminal MYB/SANT domain; however, it lacks the overall domain architecture characteristic of CCA1 or LHY; a similar pattern was observed across Streptophyta. The putative homolog identified for PSEUDO RESPONSE REGULATORs (PRRs) retains only the C‐terminal CONSTANS, CONSTANS‐like, and TOC1 (CCT) domain and lacks the N‐terminal receiver (REC) domain characteristic of canonical PRRs. Moreover, the *Chara* protein does not contain the B‐box zinc‐finger motif present in its closest *A. thaliana* homolog, CONSTANS‐LIKE 16. Consequently, it cannot be classified as a canonical PRR or CO‐like transcription factor. Based on its domain architecture and reciprocal BLAST analyses, this protein is more appropriately described as a CCT‐domain protein rather than a true PRR or CO homolog. GIGANTEA (GI) was found highly conserved only in *C. braunii* and the other representatives of the late diverging group of Streptophyta (ZCC‐grade, Phragmoplastophyta) including the transcriptome of *Coleochaete irregularis* (Supplemental Table S6, only gene presence is inferred from transcriptomic data). By contrast, ZEITLUPE (ZTL) emerged earlier, with homologs found also in *Klebsormidiaceae*. In *C. braunii* , however, no canonical ZTL ortholog containing both light, oxygen, or voltage (LOV) and Kelch‐repeat domains was identified; only a Kelch‐repeat/F‐box‐containing member of the ADAGIO family, which represents a clade parallel to ZTL, was detected. Although an LOV‐bearing ZTL homolog is also expressed in *C. irregularis* , no LOV‐bearing ZTL‐like protein was detected in *C. braunii* , where we found PHOTOTROPIN as the only LOV‐domain‐containing protein. In contrast, the subunits of the evening complex, EARLY FLOWERING 3, 4 (ELF3 and ELF4) and LUX are well conserved in *C. braunii* . While ELF4 and LUX are broadly conserved among Streptophyta, ELF3 appears to emerge later in streptophyte evolution, with the first partial homologs found in *Klebsormidiophyceae*.

**FIGURE 7:**
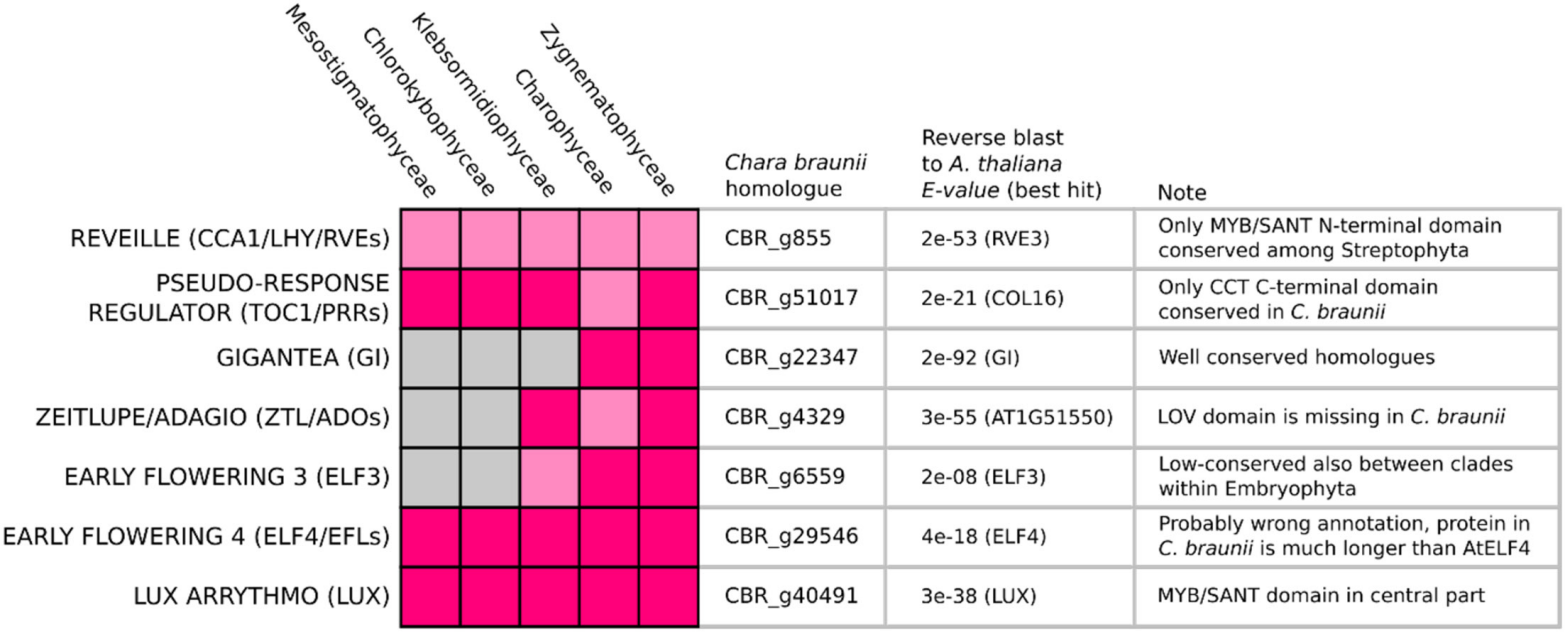
Conservation of circadian‐clock players in *Chara braunii* and the other lineages of Streptophyta. Proteomes of fully sequenced species in the Phycocosm database were searched by representative *Arabidopsis thaliana* proteins. Presence of well‐conserved homologs is marked by dark magenta, partial or distant homologs are marked by lighter, missing are gray.

## 4 Discussion

### 4.1 Rhythmic Oscillations in Branchlet Angle Are Consistent With Endogenous Regulation in *C. braunii*

*C. braunii* exhibits rhythmic apical branchlet movements resembling nyctinastic movements already described by Darwin and Darwin (1880). The rhythmic movement observed in this study was restricted to the apical whorl. Nightly closure of the apical branchlets may protect the meristem‐like thallus tip (Schubert, Holzhausen, et al. 2024) from grazing and/or epiphytic colonization during periods when light‐dependent defense mechanisms are inactive (Berger et al. 1995). In addition, Schneider et al. (2006) reported that only elongating branches responded to light intensity and suggested that this response may be mediated by blue‐light receptors such as cryptochromes or phototropins. Under CRL, branchlet movement persisted only during the first projected subjective night after transfer and was no longer detectable thereafter. However, rhythms that persist under CDL can be masked or suppressed at elevated irradiance, as shown for Arabidopsis, where continuous light dampens rhythmic outputs despite an intact clock (Niwa et al. 2009; Velez‐Ramirez et al. 2014). Similar light‐intensity‐dependent effects on rhythmic outputs have been reported in marine algae. In *Porphyra umbilicalis* , circadian growth rhythmicity was disrupted under continuous blue light even at irradiances as low as 5 μmol photons m^−2^ s^−1^ (Lüning 2001), while in *Kappaphycus alvarezii*, rhythm amplitude and period length of photosynthetic oxygen evolution varied systematically with irradiance (Granbom et al. 2001). In *C. braunii* , the attenuation of branchlet‐angle oscillations under CRL may similarly reflect irradiance‐dependent masking or damping of the observable rhythmic output. Under low irradiance (CDL), a sufficient light gradient may persist between the upper and lower sides of the branchlet. This gradient could maintain the differential elongation proposed by Schneider et al. (2006) and thereby sustain rhythmic bending. Under higher irradiance (CRL), however, light may penetrate more effectively through the chloroplast layer. As a result, the contrast in light availability between both sides of the branchlet may be reduced. This loss of asymmetry could lead to more uniform growth and, consequently, to the disappearance of rhythmic movement. However, branchlet angle is a geometric readout that cannot distinguish growth‐driven changes caused by differential cell elongation from reversible, turgor‐driven changes in position. Both processes may contribute to the observed oscillation.

Internodal cell growth did not show strong day‐night variation under light–dark cycles and remained largely constant under CRL. However, under CDL, growth was enhanced during the first projected subjective night (T12) and declined with time (T36 and T60). Under the entrained light/dark cycle, growth rates did not differ between day and night, suggesting that cell elongation in *C. braunii* is not strictly light‐dependent. This contrasts with dicotyledonous species such as *Nicotiana tabacum* , *Ricinus communis* , and Arabidopsis, where growth typically peaks at the end of the night or early in the day (Farré 2012). Under continuous light, this pattern persists, albeit shifted into subjective day phases (Poire et al. 2010; Walter et al. 2002; Walter and Schurr 2005; Wiese et al. 2007).

### 4.2 Pigment Content Reflects Irradiance Acclimation, While Photosynthetic Parameters Show no Detectable Temporal Variation

In contrast to the pigment parameters, chlorophyll fluorescence parameters, including maximum electron transport rate (ETR_max_), initial slope (α), light saturation point (I_*k*_), and dark‐adapted PSII quantum yield (Y_dark_ (II)) did not show detectable temporal variation across time points or days under either CDL or CRL (Figures S3 and S4). This absence of rhythmicity contrasts with observations in the red alga *Kappaphycus alvarezii*, where rhythmic cycles in oxygen evolution persist under constant conditions (Granbom et al. 2001). Similarly, in *Phaseolus vulgaris* , photosynthesis and leaf movement are jointly regulated by circadian rhythms (Somers 1999). However, high light can suppress these rhythms in Arabidopsis, consistent with our branchlet movement pattern and growth rate under CRL (Somers 1999; Velez‐Ramirez et al. 2011). In other lineages photosynthesis often responds directly to light rather than rhythmic cues, with few species exhibiting rhythmicity under constant light (Aros‐Mualin et al. 2023). Rhythmic regulation of photosynthesis may thus be process‐specific; for instance, net CO_2_ assimilation can fluctuate while RubisCO activation remains constant (Fredeen et al. 1991). As photosynthetic parameters did not show detectable rhythmicity under the present conditions, further investigation is needed to identify which downstream processes in *C. braunii* may be subject to endogenous temporal control.

Our pigment data suggest that pigment synthesis may be one such process. While photosynthetic parameters showed no detectable diel or between‐day variation, pigment concentrations diverged after 36 h, with consistently higher levels under CDL. This pattern likely reflects acclimation to low light, enhancing light‐harvesting capacity (Lichtenthaler et al. 2007). The accumulation of photosynthetic pigments under CDL thus indicates that *C. braunii* actively adjusts its light‐harvesting machinery in response to irradiance, even in the absence of a light–dark cycle.

Photoprotective responses such as xanthophyll cycling and nonphotochemical‐quenching (NPQ), which rely on carotenoid turnover, may also be engaged under high irradiance conditions (Cazzonelli 2011). Remarkably, the absolute concentrations of chlorophyll *a*, chlorophyll *b*, and carotenoids did not differ between treatments at T12, although the chlorophyll *a*/chlorophyll *b* ratio was already different. This likely reflects acclimation kinetics rather than absent regulation, as salt‐stress acclimation in charophytes took around 24 h (Heß et al. 2023). In addition, photosynthetic and transcriptomic responses in *C. braunii* have been reported to occur delayed (Heise et al. 2024), suggesting that a 24‐h window may be insufficient to capture full pigment reprogramming. Both processes became disrupted by the second projected subjective night in CRL. Under CDL, pigment levels declined again by T60. Similarly, in *Phaeodactylum tricornutum* , pigment‐related gene expression is modulated by both light quality and circadian regulation (Madhuri et al. 2024), pointing to a general principle of light‐dependent but clock‐modulated pigment control in algae.

### 4.3 Metabolite Accumulation Patterns Differ Between Light Intensities Under Continuous Illumination

Metabolite profiling revealed clear treatment‐specific differences in *C. braunii* . Under CDL, metabolite levels increased steadily to T60, reflecting enhanced anabolic activity and a reduced catabolic drain. In contrast, CRL caused only a transient rise followed by a decline by T60, indicating a downregulation of central metabolism (Figure 5). This pattern is consistent with irradiance‐dependent metabolic adjustment as reported in Arabidopsis (Jänkänpää et al. 2012) and other photosynthetic organisms under prolonged illumination (Proietti et al. 2021). Continuous light disrupts starch and sugar rhythms (Haque et al. 2015); these differences likely reflect differential metabolic adjustment to light intensity in *C. braunii* . They may further indicate altered coupling between the CBB and downstream carbon metabolism under CRL, resulting in reduced carbon flux into the TCA cycle and associated biosynthetic pathways. The persistent accumulation of amino and organic acids under CDL, compared with their transient increase under CRL, suggests an altered carbon‐nitrogen balance at higher irradiance (Nunes‐Nesi et al. 2010; Proietti et al. 2021). Such divergence is consistent with altered coordination of carbon and nitrogen metabolism under different irradiances (Flis et al. 2016) and mirrors the broader pattern by which continuous light decouples metabolic outputs from endogenous temporal control, a process already described for photosynthetic organisms such as *Chara* (Kwiatkowska et al. 1995) and higher plants (Dodd et al. 2015).

The lower malate and fumarate levels under CRL suggest altered carbon metabolism under higher irradiance. The decline in pyruvate under CRL may thus reflect reduced glycolytic input into the TCA cycle or diversion into stress‐related pathways (Hildebrandt et al. 2015; Plaxton and Podestá 2006), consistent with a metabolic shift away from anabolic processes under higher irradiance. In parallel, increased serine and phenylalanine under CRL may indicate metabolic stress responses (de Vries et al. 2020; Ho and Saito 2001; Rieseberg et al. 2023; Ros et al. 2014). The serine rise likely results from enhanced glycine‐to‐serine conversion, a hallmark of intensified photorespiration (Bauwe et al. 2010; Timm and Bauwe 2013). Elevated α‐aminobutyrate at T36 and T60 represents an additional treatment‐specific response whose functional significance in *C. braunii* remains unresolved. Because branchlet movement and metabolite profiles were assessed at different temporal resolutions, their temporal relationship cannot be evaluated directly. Nevertheless, the co‐occurrence of attenuated branchlet‐angle oscillations and a distinct metabolic profile under CRL suggests that higher irradiance is associated with changes in both rhythmic outputs and physiological acclimation. Whether these two processes are causally linked or represent independent responses to the same environmental variable cannot be resolved from the present data, but their co‐occurrence provides a framework for future mechanistic investigation.

### 4.4 Clock‐Associated Gene Complements in *C. braunii* Differ From Flowering Plants

Despite the importance of circadian clock genes, a detailed study of their evolution and function in the streptophyte lineage is still lacking, except for the relatively early‐diverging lineage represented by *Klebsormidium* (Linde et al. 2017). Reconstruction of the origin of circadian regulatory genes is complicated by their modular nature, as the presence of a single domain (typically DNA‐binding) does not provide evidence for the conservation of the full protein and its physiological role. This is particularly evident in the CCA1/LHY/RVE family, whose members are poorly conserved across streptophytes, with the exception of their MYB/SANT domain. In contrast, the TOC1/PRR family is relatively well conserved, except in *C. braunii* , where no canonical TOC1 homolog was identified. The Evening Complex components appear to be conserved across several streptophyte lineages, except for the ELF3 component, which, consistent with Linde et al. (2017), we first identified in *Klebsormidium*, along with ZTL homologs. The interaction of ZTL with GI, however, may have arisen later, as GI appears only in the ZCC grade (Zygnematophyceae, Coleochaophyceae, and Charophyceae). Whether this interaction occurs in *C. braunii* remains uncertain because only a ZTL/ADO homolog lacking the photoreceptive LOV domain was identified. Consequently, the light‐responsive regulatory mechanisms in *C. braunii* are likely to differ from those characterized in flowering plants.

## 5 Conclusions

Our findings demonstrate that *C. braunii* exhibits circadian oscillations in branchlet angle that persist under low‐irradiance continuous light but are attenuated at higher irradiance. Internodal growth showed more limited temporal structure under the applied conditions. This light‐intensity‐dependent modulation is consistent with the operation of an endogenous regulatory component. Pigment content and metabolite profiles reflected irradiance‐dependent acclimation over time, including transiently higher pigment concentrations and increased levels of selected TCA‐cycle intermediates under CDL. These findings provide a basis for future studies investigating whether these parameters are additionally subject to temporal regulation. The identification of several clock‐associated gene families in *C. braunii* , albeit with incomplete conservation relative to flowering plants, provides a molecular framework for functional experiments aimed at resolving whether these components contribute to the observed rhythmic outputs and their attenuation under higher irradiance.

## Author Contributions

K.K. conceptualized the study, performed the growth and movement experiment, and conducted sampling with *C. braunii* . A.‐M.M. carried out photosynthesis measurements and pigment analyses. A.‐M.M., C.M.H., and S.T. performed and evaluated metabolite measurements. S.V. conducted all phylogenetic analyses. J.P. and H.S. supervised the evaluation of the experiments. K.K., S.V., C.M.H., and A.‐M.M. drafted the manuscript, and all authors contributed to writing and approving the final version.

## Funding

This work was supported by HBFG Programme, (GZ: INST 264/125‐1 FUGG) and Detsche Forschungsgemeinschaft(HA2002/25‐1 and SCHU983/24‐1).

## Conflicts of Interest

The authors declare no conflicts of interest.

## Supporting information




**Figure S1:** Spectral composition of the aquarium lighting (yellow) and the LED spectra in the custom‐built cultivation box under dim light (DL, blue) and regular light (RL, green) conditions, used for *Chara braunii* experiments following precultivation.
**Figure S2:** Timelapse imaging of *C. braunii* top branchlets under continuous light over 3 nights and 3 days. The dark blue band corresponds to image taken at midnight and the light blue to images taken at noon. (A–F) Continuous regular light (CRL, 30 μmol photons m^−2^ s^−1^)—branchlets open the first day and stay open until the end of experiment. (G–L) continuous dim light (CDL, 15 μmol photons m^−2^ s^−1^)—branchlets open and close in a diurnal rhythm.
**Figure S3:** Photosynthetic performance of *C. braunii* cultivated under continuous dim light (CDL, 15 μmol photons m^−2^ s^−1^, yellow) and continuous regular light (CRL, 30 μmol photons m^−2^ s^−1^, orange). Samples were measured after the first (D1), second (D2), and third night (D3) at 6:00, 13:00, and 20:00. Shown are (A) the maximum electron transport rate (ETR_max_) (B) the initial slope (α), and (C) the light saturation point (I_k_) of the photosynthesis–irradiance curve. Data represent raw values (*n* = 2). No measurements were taken at D2 13:00.
**Figure S4:** Dark yield (Y_dark_ (II)) of *C. braunii* cultivated under continuous dim light (CDL, 15 μmol photons m^−2^ s^−1^, yellow) and continuous regular light (CRL, 30 μmol photons m^−2^ s^−1^, orange). Samples were measured after the first (D1), second (D2), and third night (D3) at 6:00, 13:00, and 20:00. Data represent raw values (*n* = 2). No measurements were taken at D2 13:00.
**Table S1:** Metabolites identified in *C. braunii* in this study.

**Table S2:** Quantified metabolites in *Chara braunii* metabolites.
**Table S3:** Sequences of canonical circadian‐clock proteins from *Arabidopsis thaliana* used as queries for BLAST searches in *C. braunii* .
**Table S4:** Replicate‐level JTK_CYCLE results and FDR‐adjusted *p* values for branchlet angle time series under the different light conditions.
**Table S5:** Statistical results for pigment comparisons between continuous dim light (CDL) and continuous regular light (CRL) at T12, T36, and T60.
**Table S6:** Statistical results for individual metabolite comparisons between T12 and the later sampling times T36 and T60 within each light treatment.

**Video S1:** Time‐lapse imaging of branchlet angle dynamics in *C. braunii* under continuous light conditions at Day 1.

**Video S2:** Time‐lapse imaging of branchlet angle dynamics in *C. braunii* under continuous light conditions at Day 2.

**Video S3:** Time‐lapse imaging of branchlet angle dynamics in *C. braunii* under continuous light conditions at Day 3.

## Data Availability

The data that supports the findings of this study are available in the Supporting Information of this article.

## References

[pld370194-bib-0001] Alabadí, D. , T. Oyama , M. J. Yanovsky , F. G. Harmon , P. Más , and S. A. Kay . 2001. “Reciprocal Regulation Between TOC1 and LHY/CCA1 Within the Arabidopsis Circadian Clock.” Science 293, no. 5531: 880–883. 10.1126/science.1061320 11486091

[pld370194-bib-0002] Aros‐Mualin, D. , C. R. Guadagno , D. Silvestro , and M. Kessler . 2023. “Light, Rather Than Circadian Rhythm, Regulates Gas Exchange in Ferns and Lycophytes.” Plant Physiology 191, no. 3: 1634–1647. 10.1093/plphys/kiad036 PMC10022864 36691320

[pld370194-bib-0003] Bates, D. , M. Mächler , B. Bolker , and S. Walker . 2015. “Fitting Linear Mixed‐Effects Models Using lme4.” Journal of Statistical Software 67, no. 1: 1–48. 10.18637/jss.v067.i01.

[pld370194-bib-0004] Bauwe, H. , M. Hagemann , and A. R. Fernie . 2010. “Photorespiration: Players, Partners and Origin.” Trends in Plant Science 15, no. 6: 330–336. 10.1016/j.tplants.2010.03.006 20403720

[pld370194-bib-0005] Beilby, M. J. , C. E. Turi , T. C. Baker , F. J. M. Tymm , and S. J. Murch . 2015. “Circadian Changes in Endogenous Concentrations of Indole‐3‐Acetic Acid, Melatonin, Serotonin, Abscisic Acid and Jasmonic Acid in Characeae (*Chara australis* Brown).” Plant Signaling & Behavior 10, no. 11: e1082697. 10.1080/15592324.2015.1082697 PMC4883837 26382914

[pld370194-bib-0006] Berger, S. , E. Bell , A. Sadka , and J. E. Mullet . 1995. “ *Arabidopsis Thaliana* Atvsp Is Homologous to Soybean VspA and VspB, Genes Encoding Vegetative Storage Protein Acid Phosphatases, and Is Regulated Similarly by Methyl Jasmonate, Wounding, Sugars, Light and Phosphate.” Plant Molecular Biology 27, no. 5: 933–942. 10.1007/BF00037021 7766883

[pld370194-bib-0007] Bulut, M. , E. Karakas , and A. R. Fernie . 2025. “Adjustments of Plant Primary Metabolism in the Face of Climate Change.” Journal of Experimental Botany 76: 4804–4816. 10.1093/jxb/eraf116 PMC12587424 40071778

[pld370194-bib-0008] Bünning, E. 1936. “Die endonome Tagesrhythmik als Grundlage der photoperiodischen Reaktion.” Berichte der Deutschen Botanischen Gesellschaft 54, no. 10: 590–607.

[pld370194-bib-0009] Camacho, C. , G. M. Boratyn , V. Joukov , R. Vera Alvarez , and T. L. Madden . 2023. “ElasticBLAST: Accelerating Sequence Search via Cloud Computing.” BMC Bioinformatics 24, no. 1: 117. 10.1186/s12859-023-05245-9 PMC10040096 36967390

[pld370194-bib-0010] Cazzonelli, C. I. 2011. “Carotenoids in Nature: Insights From Plants and Beyond.” Functional Plant Biology 38, no. 11: 833–847. 10.1071/FP11192 32480941

[pld370194-bib-0012] Cha, J.‐Y. , J. Kim , T.‐S. Kim , et al. 2017. “GIGANTEA Is a Co‐Chaperone Which Facilitates Maturation of ZEITLUPE in the Arabidopsis Circadian Clock.” Nature Communications 8, no. 1: 3. 10.1038/s41467-016-0014-9 PMC5431898 28232745

[pld370194-bib-0013] Darwin, C. , and F. E. Darwin . 1880. The Power of Movement in Plants. John Murray.

[pld370194-bib-0014] de Vries, J. , S. de Vries , B. A. Curtis , et al. 2020. “Heat Stress Response in the Closest Algal Relatives of Land Plants Reveals Conserved Stress Signaling Circuits.” Plant Journal 103, no. 3: 1025–1048. 10.1111/tpj.14782 32333477

[pld370194-bib-0015] Dodd, A. N. , F. E. Belbin , A. Frank , and A. A. R. Webb . 2015. “Interactions Between Circadian Clocks and Photosynthesis for the Temporal and Spatial Coordination of Metabolism.” Frontiers in Plant Science 6: 245. 10.3389/fpls.2015.00245 PMC4391236 25914715

[pld370194-bib-0016] Durand, M. , X. Zhuang , Y. Salmon , and T. M. Robson . 2024. “Caught Between Two States: The Compromise in Acclimation of Photosynthesis, Transpiration and Mesophyll Conductance to Different Amplitudes of Fluctuating Irradiance.” Plant, Cell & Environment 47, no. 12: 5220–5236. 10.1111/pce.15107 39169893

[pld370194-bib-0017] Eilers, P. H. , and J. C. Peeters . 1988. “A Model for the Relationship Between Light Intensity and the Rate of Photosynthesis in Phytoplankton.” Ecological Modelling 42, no. 3–4: 199–215.

[pld370194-bib-0018] Elzhov, T. , K. M. Mullen , A.‐N. Spiess , and B. Bolker . 2023. “minpack. ln: R Interface to the Levenberg‐Marquardt Nonlinear Least‐Squares Algorithm Found in MINPACK.” Plus Support for Bounds. Disponível em: https://cran.rproject.org/web/packages/minpack.lm/minpack.lm.pdf.

[pld370194-bib-0019] Farré, E. M. 2012. “The Regulation of Plant Growth by the Circadian Clock.” Plant Biology 14, no. 3: 401–410. 10.1111/j.1438-8677.2011.00548.x 22284304

[pld370194-bib-0020] Flis, A. , R. Sulpice , D. D. Seaton , et al. 2016. “Photoperiod‐Dependent Changes in the Phase of Core Clock Transcripts and Global Transcriptional Outputs at Dawn and Dusk in Arabidopsis.” Plant, Cell & Environment 39, no. 9: 1955–1981. 10.1111/pce.12754 27075884

[pld370194-bib-0021] Fredeen, A. L. , T. L. Hennessey , and C. B. Field . 1991. “Biochemical Correlates of the Circadian Rhythm in Photosynthesis in *Phaseolus Vulgaris* .” Plant Physiology 97, no. 1: 415–419. 10.1104/pp.97.1.415 PMC1081014 16668402

[pld370194-bib-0022] Globig, S. , I. Rosen , and H. W. Janes . 1997. “Continuous Light Effects on Photosynthesis and Carbon Metabolism in Tomato.” Acta Horticulturae 418: 141–152. 10.17660/ActaHortic.1997.418.19.

[pld370194-bib-0023] Granbom, M. , M. Pedersén , P. Kadel , and K. Lüning . 2001. “Circadian Rhythm of Photosynthetic Oxygen Evolution in *Kappaphycus alvarezii* (Rhodophyta): Dependence on Light Quantity and Quality.” Journal of Phycology 37, no. 6: 1020–1025.

[pld370194-bib-0024] Green, P. B. 1954. “The Spiral Growth Pattern of the Cell Wall in *Nitella Axillaris* .” American Journal of Botany 41: 403–409.

[pld370194-bib-0025] Grigoriev, I. V. , R. D. Hayes , S. Calhoun , et al. 2021. “PhycoCosm, a Comparative Algal Genomics Resource.” Nucleic Acids Research 49, no. D1: D1004–D1011. 10.1093/nar/gkaa898 PMC7779022 33104790

[pld370194-bib-0026] Haque, M. S. , K. H. Kjaer , E. Rosenqvist , and C.‐O. Ottosen . 2015. “Continuous Light Increases Growth, Daily Carbon Gain, Antioxidants, and Alters Carbohydrate Metabolism in a Cultivated and a Wild Tomato Species.” Frontiers in Plant Science 6: 522. 10.3389/fpls.2015.00522 PMC4499675 26217371

[pld370194-bib-0027] Heise, C. M. , M. Hagemann , and H. Schubert . 2024. “Photosynthetic Response of *Chara braunii* Towards Different Bicarbonate Concentrations.” Physiologia Plantarum 176, no. 2: e14234. 10.1111/ppl.14234 38439180

[pld370194-bib-0028] Heß, D. , C. M. Heise , H. Schubert , W. R. Hess , and M. Hagemann . 2023. “The Impact of Salt Stress on the Physiology and the Transcriptome of the Model Streptophyte Green Alga *Chara braunii* .” Physiologia Plantarum 175, no. 6: e14123. 10.1111/ppl.14123 38148211

[pld370194-bib-0029] Hildebrandt, T. M. , A. N. Nesi , W. L. Araújo , and H.‐P. Braun . 2015. “Amino Acid Catabolism in Plants.” Molecular Plant 8, no. 11: 1563–1579. 10.1016/j.molp.2015.09.005 26384576

[pld370194-bib-0030] Ho, C.‐L. , and K. Saito . 2001. “Molecular Biology of the Plastidic Phosphorylated Serine Biosynthetic Pathway in *Arabidopsis Thaliana* .” Amino Acids 20, no. 3: 243–259. 10.1007/s007260170042 11354602

[pld370194-bib-0031] Holm, K. , T. Källman , N. Gyllenstrand , H. Hedman , and U. Lagercrantz . 2010. “Does the Core Circadian Clock in the Moss *Physcomitrella patens* (Bryophyta) Comprise a Single Loop?” BMC Plant Biology 10, no. 1: 109. 10.1186/1471-2229-10-109 PMC3017809 20550695

[pld370194-bib-0032] Hothorn, T. , F. Bretz , and P. Westfall . 2008. “Simultaneous Inference in General Parametric Models.” Biometrical Journal 50, no. 3: 346–363. 10.1002/bimj.200810425 18481363

[pld370194-bib-0033] Hughes, M. E. , J. B. Hogenesch , and K. Kornacker . 2010. “JTK_CYCLE: An Efficient Nonparametric Algorithm for Detecting Rhythmic Components in Genome‐Scale Data Sets.” Journal of Biological Rhythms 25, no. 5: 372–380. 10.1177/0748730410379711 PMC3119870 20876817

[pld370194-bib-0034] Jänkänpää, H. J. , Y. Mishra , W. P. Schröder , and S. Jansson . 2012. “Metabolic Profiling Reveals Metabolic Shifts in Arabidopsis Plants Grown Under Different Light Conditions.” Plant, Cell & Environment 35, no. 10: 1824–1836. 10.1111/j.1365-3040.2012.02519.x 22497620

[pld370194-bib-0035] Kanehisa, M. , and S. Goto . 2000. “KEGG: Kyoto Encyclopedia of Genes and Genomes.” Nucleic Acids Research 28, no. 1: 27–30. 10.1093/nar/28.1.27 PMC102409 10592173

[pld370194-bib-0036] Kolde, R. 2019. “Pheatmap: Pretty Heatmaps.” R Package Version, 1 2 726.

[pld370194-bib-0037] Kurtović, K. , J. Petrášek , and S. Vosolsobě . 2026. “Optimized Laboratory Maintenance and Functional Testing of Chara Braunii.” Current Protocols 6, no. 1: e70279. 10.1002/cpz1.70279 PMC12750483 41467534

[pld370194-bib-0038] Kurtović, K. , S. Vosolsobě , D. Nedvěd , et al. 2025. “The Role of Indole‐3‐Acetic Acid and Characterization of PIN Transporters in Complex Streptophyte Alga *Chara braunii* .” New Phytologist 246, no. 3: 1066–1083. 10.1111/nph.70019 PMC11982790 40047465

[pld370194-bib-0039] Kwiatkowska, M. , K. Poptońska , A. a. Gosek , and S. Malinowski . 1995. “Circadian Rhythm of Protein Synthesis in Generative and Non‐Generative Antheridial Cells of *Chara vulgaris* L.” Biological Rhythm Research 26, no. 1: 55–67.

[pld370194-bib-0040] Lazar, D. , S. J. Murch , M. J. Beilby , and S. Al Khazaaly . 2013. “Exogenous Melatonin Affects Photosynthesis in characeae *Chara australis* .” Plant Signaling & Behavior 8, no. 3: e23279. 10.4161/psb.23279 PMC3676496 23299331

[pld370194-bib-0041] Leebens‐Mack, J. H. , M. S. Barker , E. J. Carpenter , et al. 2019. “One Thousand Plant Transcriptomes and the Phylogenomics of Green Plants.” Nature 574, no. 7780: 679–685. 10.1038/s41586-019-1693-2 PMC6872490 31645766

[pld370194-bib-0042] Lenth, R. 2023. “Emmeans: Estimated Marginal Means, aka Least‐Squares Means_', R Package Version 1.” R Package Version 1.8. 5.

[pld370194-bib-0043] Lichtenthaler, H. K. , F. Babani , and G. Langsdorf . 2007. “Chlorophyll Fluorescence Imaging of Photosynthetic Activity in Sun and Shade Leaves of Trees.” Photosynthesis Research 93, no. 1: 235–244. 10.1007/s11120-007-9174-0 17486425

[pld370194-bib-0044] Linde, A.‐M. , D. M. Eklund , A. Kubota , et al. 2017. “Early Evolution of the Land Plant Circadian Clock.” New Phytologist 216, no. 2: 576–590. 10.1111/nph.14487 PMC5638080 28244104

[pld370194-bib-0045] Lüning, K. 2001. “Circadian Growth in *Porphyra Umbilicalis* (Rhodophyta): Spectral Sensitivity of the Circadian System.” Journal of Phycology 37, no. 1: 52–58.

[pld370194-bib-0046] Lüning, K. , E. Titlyanov , and T. Titlyanova . 1997. “Diurnal and Circadian Periodicity of Mitosis and Growth in Marine Macroalgae. III. The Red Alga *Porphyra Umbilicalis* .” European Journal of Phycology 32, no. 2: 167–173.

[pld370194-bib-0047] Madhuri, S. , B. Lepetit , A. H. Fürst , and P. G. Kroth . 2024. “A Knockout of the Photoreceptor PtAureo1a Results in Altered Diel Expression of Diatom Clock Components.” Plants 13, no. 11: 1465. 10.3390/plants13111465 PMC11174801 38891274

[pld370194-bib-0048] Maidel, A.‐M. , and H. Schubert . 2026. “Morphological and Physiological Traits of Two Closely Related Charophyte Species: *Chara virgata* Kütz. and *C. globularis* Thuill.” Aquatic Botany 202: 103929.

[pld370194-bib-0049] Más, P. , W.‐Y. Kim , D. E. Somers , and S. A. Kay . 2003. “Targeted Degradation of TOC1 by ZTL Modulates Circadian Function in *Arabidopsis thaliana* .” 426, no. 6966, pp. 567–570. 10.1038/nature02163 14654842

[pld370194-bib-0050] Nishiyama, T. , H. Sakayama , J. de Vries , et al. 2018. “The Chara Genome: Secondary Complexity and Implications for Plant Terrestrialization.” Cell 174, no. 2: 448–464. 10.1016/j.cell.2018.06.033 30007417

[pld370194-bib-0051] Niwa, Y. , T. Yamashino , and T. Mizuno . 2009. “The Circadian Clock Regulates the Photoperiodic Response of Hypocotyl Elongation Through a Coincidence Mechanism in *Arabidopsis Thaliana* .” Plant and Cell Physiology 50, no. 4: 838–854. 10.1093/pcp/pcp028 19233867

[pld370194-bib-0052] Nunes‐Nesi, A. , A. R. Fernie , and M. Stitt . 2010. “Metabolic and Signaling Aspects Underpinning the Regulation of Plant Carbon Nitrogen Interactions.” Molecular Plant 3, no. 6: 973–996. 10.1093/mp/ssq049 20926550

[pld370194-bib-0053] Nusinow, D. A. , A. Helfer , E. E. Hamilton , et al. 2011. “The ELF4–ELF3–LUX Complex Links the Circadian Clock to Diurnal Control of Hypocotyl Growth.” Nature 475, no. 7356: 398–402. 10.1038/nature10182 PMC3155984 21753751

[pld370194-bib-0054] Oksanen, J. , F. G. Blanchet , R. Kindt . et al. 2013. “Community Ecology Package.” R Package Version, 2. 321–326.

[pld370194-bib-0055] Plaxton, W. C. , and F. E. Podestá . 2006. “The Functional Organization and Control of Plant Respiration.” Critical Reviews in Plant Sciences 25, no. 2: 159–198.

[pld370194-bib-0056] Poire, R. , A. Wiese‐Klinkenberg , B. Parent , et al. 2010. “Diel Time‐Courses of Leaf Growth in Monocot and Dicot Species: Endogenous Rhythms and Temperature Effects.” Journal of Experimental Botany 61, no. 6: 1751–1759. 10.1093/jxb/erq049 PMC2852670 20299442

[pld370194-bib-0057] Proietti, S. , S. Moscatello , F. Riccio , P. Downey , and A. Battistelli . 2021. “Continuous Lighting Promotes Plant Growth, Light Conversion Efficiency, and Nutritional Quality of *Eruca Vesicaria* (L.) Cav. in Controlled Environment With Minor Effects Due to Light Quality.” Frontiers in Plant Science 12: 730119. 10.3389/fpls.2021.730119 PMC8546256 34712255

[pld370194-bib-0058] R Core Team . 2023. “R Core Team: A Language and Environment for Statistical (4.3.1) [Computer program].” https://www.R‐project.org/.

[pld370194-bib-0059] Reinholdt, O. , S. Schwab , Y. Zhang , et al. 2019. “Redox‐Regulation of Photorespiration Through Mitochondrial Thioredoxin o1.” Plant Physiology 181, no. 2: 442–457. 10.1104/pp.19.00559 PMC6776843 31413204

[pld370194-bib-0060] Rhee, S. Y. , W. Beavis , T. Z. Berardini , et al. 2003. “The Arabidopsis Information Resource (TAIR): A Model Organism Database Providing a Centralized, Curated Gateway to Arabidopsis Biology, Research Materials and Community.” Nucleic Acids Research 31, no. 1: 224–228. 10.1093/nar/gkg076 PMC165523 12519987

[pld370194-bib-0061] Rieseberg, T. P. , A. Dadras , J. M. Fürst‐Jansen , et al. 2023. “Crossroads in the Evolution of Plant Specialized Metabolism.” Seminars in Cell & Developmental Biology 134: 37–58. 10.1016/j.semcdb.2022.03.004. 35292191

[pld370194-bib-0062] Ros, R. , J. Muñoz‐Bertomeu , and S. Krueger . 2014. “Serine in Plants: Biosynthesis, Metabolism, and Functions.” Trends in Plant Science 19, no. 9: 564–569. 10.1016/j.tplants.2014.06.003 24999240

[pld370194-bib-0063] Sarkar, D. 2008. “Multivariate Data Visualization With R.” Use R.

[pld370194-bib-0064] Schneider, S. , C. Ziegler , and A. Melzer . 2006. “Growth Towards Light as an Adaptation to High Light Conditions in *Chara* Branches.” New Phytologist 172, no. 1: 83–91. 10.1111/j.1469-8137.2006.01812.x 16945091

[pld370194-bib-0065] Schneider, S. C. , D. E. Pichler , T. Andersen , and A. Melzer . 2015. “Light Acclimation in Submerged Macrophytes: The Roles of Plant Elongation, Pigmentation and Branch Orientation Differ Among *Chara* Species.” Aquatic Botany 120: 121–128.

[pld370194-bib-0066] Schubert, H. , K.‐G. Bernhardt , M. T. Casanova , T. Gregor , W.‐H. Kusber , and B. Turner . 2024. “Taxonomic Concepts and Taxonomic Evolution in European Characeae.” In Charophytes of Europe, edited by H. Schubert , I. Blindow , E. Nat , et al. 65–99. Springer Nature Switzerland.

[pld370194-bib-0067] Schubert, H. , A. Holzhausen , P. a. Nowak , and M. T. Casanova . 2024. “Ontogenesis and Life Cycle of Charophytes.” In Charophytes of Europe, edited by H. Schubert , I. Blindow , E. Nat , et al. 13–33. Springer Nature Switzerland.

[pld370194-bib-0068] Schweiger, R. , M. C. Baier , M. a. Persicke , and C. Müller . 2014. “High Specificity in Plant Leaf Metabolic Responses to Arbuscular Mycorrhiza.” Nature Communications 5, no. 1: 3886. 10.1038/ncomms4886 24848943

[pld370194-bib-0069] Somers, D. E. 1999. “The Physiology and Molecular Bases of the Plant Circadian Clock.” Plant Physiology 121, no. 1: 9–20. 10.1104/pp.121.1.9 PMC1539225 10482655

[pld370194-bib-0071] Timm, S. , and H. Bauwe . 2013. “The Variety of Photorespiratory Phenotypes–Employing the Current Status for Future Research Directions on Photorespiration.” Plant Biology 15, no. 4: 737–747. 10.1111/j.1438-8677.2012.00691.x 23171236

[pld370194-bib-0072] Velez‐Ramirez, A. I. , W. van Ieperen , D. Vreugdenhil , and F. F. Millenaar . 2011. “Plants Under Continuous Light.” Trends in Plant Science 16, no. 6: 310–318. 10.1016/j.tplants.2011.02.003 21396878

[pld370194-bib-0073] Velez‐Ramirez, A. I. , W. van Ieperen , D. Vreugdenhil , P. M. van Poppel , E. Heuvelink , and F. F. Millenaar . 2014. “A Single Locus Confers Tolerance to Continuous Light and Allows Substantial Yield Increase in Tomato.” Nature Communications 5, no. 1: 4549. 10.1038/ncomms5549 25093373

[pld370194-bib-0074] Venkat, A. , and S. Muneer . 2022. “Role of Circadian Rhythms in Major Plant Metabolic and Signaling Pathways.” Frontiers in Plant Science 13: 836244. 10.3389/fpls.2022.836244 PMC9019581 35463437

[pld370194-bib-0075] Walter, A. , and U. Schurr . 2005. “Dynamics of Leaf and Root Growth: Endogenous Control Versus Environmental Impact.” Annals of Botany 95, no. 6: 891–900. 10.1093/aob/mci103 PMC4246750 15767269

[pld370194-bib-0076] Walter, A. , H. Spies , S. Terjung , R. Küsters , N. Kirchgessner , and U. Schurr . 2002. “Spatio‐Temporal Dynamics of Expansion Growth in Roots: Automatic Quantification of Diurnal Course and Temperature Response by Digital Image Sequence Processing.” Journal of Experimental Botany 53, no. 369: 689–698. 10.1093/jexbot/53.369.689 11886889

[pld370194-bib-0077] Webb, A. A. R. 2003. “The Physiology of Circadian Rhythms in Plants.” New Phytologist 160, no. 2: 281–303. 10.1046/j.1469-8137.2003.00895.x 33832173

[pld370194-bib-0078] Wellburn, A. R. 1994. “The Spectral Determination of Chlorophylls a and b, as Well as Total Carotenoids, Using Various Solvents With Spectrophotometers of Different Resolution.” Journal of Plant Physiology 144, no. 3: 307–313.

[pld370194-bib-0079] Wickell, D. , L.‐Y. Kuo , H.‐P. Yang , et al. 2021. “Underwater CAM Photosynthesis Elucidated by *Isoetes* Genome.” Nature Communications 12, no. 1: 6348. 10.1038/s41467-021-26644-7 PMC8566536 34732722

[pld370194-bib-0080] Wiese, A. , M. M. Christ , O. Virnich , U. Schurr , and A. Walter . 2007. “Spatio‐Temporal Leaf Growth Patterns of Arabidopsis Thaliana and Evidence for Sugar Control of the Diel Leaf Growth Cycle.” New Phytologist 174, no. 4: 752–761. 10.1111/j.1469-8137.2007.02053.x 17504459

