# Supplementary material for: Circadian Oscillations in Branchlet Angle and Irradiance‐Dependent Acclimation in Chara braunii Under Continuous Light
Source: Plant Direct. 2026 Oct 5;10(10):e70194. doi: 10.1002/pld3.70194 (PMC13639390; doi:10.1002/pld3.70194)
Supplement: Supplementary file 1 [file PLD3-10-e70194-s002.docx]

***Plant Direct* Supporting Information**

Article title: **Circadian oscillations in branchlet angle and irradiance-dependent acclimation in Chara braunii under continuous light**

Authors: Alena-Maria Maidel, Katarina Kurtović, Carolin M. Heise, Stanislav Vosolsobě, Jan Petrášek, Stefan Timm, Hendrik Schubert

The following Supporting Information is available for this article:

Figure S1: Spectral composition of the aquarium lighting (yellow) and the LED spectra in the custom-built cultivation box under dim light (DL, blue) and regular light (RL, green) conditions, used for *Chara braunii* experiments following pre-cultivation.

Figure S2: Timelapse imaging of *Chara braunii* top branchlets under continuous light over 3 nights and 3 days. The dark blue band corresponds to image taken at midnight and the light blue to images taken at noon. A-F: continuous regular light (CRL, 30 µmol photons m⁻² s⁻¹) - branchlets open the first day and stay open until the end of experiment. G-L continuous dim light (CDL, 15 µmol photons m⁻² s⁻¹) - branchlets open and close in a diurnal rhythm.

Figure S3: Photosynthetic performance of *Chara braunii* cultivated under continuous dim light (CDL, 15 µmol photons m⁻² s⁻¹, yellow) and continuous regular light (CRL, 30 µmol photons m⁻² s⁻¹, orange). Samples were measured after the first (D1), second (D2), and third night (D3) at 6:00, 13:00, and 20:00. Shown are (A) the maximum electron transport rate (ETR_max_) (B) the initial slope (α), and (C) the light saturation point (I_k_) of the photosynthesis–irradiance curve. Data represent raw values (n = 2). No measurements were taken at D2 13:00.

Figure S4: Dark Yield (Y_dark_(II)) of *Chara braunii* cultivated under continuous dim light (CDL, 15 µmol photons m⁻² s⁻¹, yellow) and continuous regular light (CRL, 30 µmol photons m⁻² s⁻¹, orange). Samples were measured after the first (D1), second (D2), and third night (D3) at 6:00, 13:00, and 20:00. Data represent raw values (n = 2). No measurements were taken at D2 13:00.

Table S1: Metabolites identified in *Chara braunii* in this study.

Supplementary Table S2: Quantified Metabolites in *Chara braunii* Metabolites

Supplementary Table S3: Sequences of canonical circadian-clock proteins from *Arabidopsis thaliana* used as queries for BLAST searches in *Chara braunii*.

Supplementary Table S4: Replicate-level JTK_CYCLE results and FDR-adjusted p-values for branchlet angle time series under the different light conditions.

Supplementary Table S5: Statistical results for pigment comparisons between continuous dim light (CDL) and continuous regular light (CRL) at T12, T36, and T60.

Supplementary Table S6: Statistical results for individual metabolite comparisons between T12 and the later sampling times T36 and T60 within each light treatment.

Supplementary Video S1: Time-lapse imaging of branchlet angle dynamics in *Chara braunii* under continuous light conditions at Day 1.

Supplementary Video S2: Time-lapse imaging of branchlet angle dynamics in *Chara braunii* under continuous light conditions at Day 2.

Supplementary Video S3: Time-lapse imaging of branchlet angle dynamics in *Chara braunii* under continuous light conditions at Day 3.

Supplementary Tables S2-S6 can be found in the separate Excel file (Supplemental_Tables.xlsx)

Supplementary Video S1, S2 and S3 can be found in the separate Video files (Day_X_scalebar.avi); X = 1, 2 or 3.

**Fig. S1:** Spectral composition of the aquarium lighting (yellow) and the LED spectra in the custom-built cultivation box under dim light (DL, blue) and regular light (RL, green) conditions, used for *Chara braunii* experiments following pre-cultivation.

**Fig. S2:** Timelapse imaging of *Chara braunii* top branchlets under continuous light over 3 nights and 3 days. The dark blue band corresponds to image taken at midnight and the light blue to images taken at noon. A-F: continuous regular light (CRL, 30 µmol photons m⁻² s⁻¹) - branchlets open the first day and stay open until the end of experiment. G-L continuous dim light (CDL, 15 µmol photons m⁻² s⁻¹) - branchlets open and close in a diurnal rhythm.

**Fig. S3:** Photosynthetic performance of *Chara braunii* cultivated under continuous dim light (CDL, 15 µmol photons m⁻² s⁻¹, yellow) and continuous regular light (CRL, 30 µmol photons m⁻² s⁻¹, orange). Samples were measured after the first (D1), second (D2), and third night (D3) at 6:00, 13:00, and 20:00. Shown are (A) the maximum electron transport rate (ETR_max_) (B) the initial slope (α), and (C) the light saturation point (I_k_) of the photosynthesis–irradiance curve. Data represent raw values (n = 2). No measurements were taken at D2 13:00.

**Fig. S4:** Dark Yield (Y_dark_(II)) of *Chara braunii* cultivated under continuous dim light (CDL, 15 µmol photons m⁻² s⁻¹, yellow) and continuous regular light (CRL, 30 µmol photons m⁻² s⁻¹, orange). Samples were measured after the first (D1), second (D2), and third night (D3) at 6:00, 13:00, and 20:00. Data represent raw values (n = 2). No measurements were taken at D2 13:00.

**Table S1:** Metabolites identified in *Chara braunii* in this study.

| **Metabolite** | **Abbreviation** |
| --- | --- |
| ***Organic acids*** | X |
| Succinate | X |
| Malate | X |
| Citrate + Isocitrate | X |
| Fumarate | X |
| *cis*-Aconitate | X |
| Lactate | X |
| Citrulline | CIT |
| α -aminobutyrate | X |
| ***Amino acids*** | X |
| Asparagine | ASN |
| Aspartate | ASP |
| Glutamate | GLU |
| Serine | SER |
| Glutamine | GLN |
| Histidine | HIS |
| Glycine | GLY |
| Threonine | THR |
| γ-aminobutyrate | GABA |
| Tyrosine | TYR |
| Valine | VAL |
| Methionine | MET |
| Tryptophan | TRP |
| Phenylalanine | PHE |
| Isoleucine | ILE |
| Leucine | LEU |
| Lysine | LYS |
| Proline | PRO |
| Ornitine | ORN |
| Arginine | ARG |
| ***Modified amino acids*** |  |
| Hydroxyproline | HYP |
| ***Nuleoside / Vitamin derivatives*** |  |
| Adenosine | X |
| Nicotinate | X |
| ***Carbohydrate intermediates*** |  |
| 3-Phosphoglycerate | 3-PGA |

Metabolites and abbreviations were identified via comparison of peak characteristics with reference standards.
